# The Global Regulatory Architecture of Transcription during the *Caulobacter* Cell Cycle

**DOI:** 10.1371/journal.pgen.1004831

**Published:** 2015-01-08

**Authors:** Bo Zhou, Jared M. Schrader, Virginia S. Kalogeraki, Eduardo Abeliuk, Cong B. Dinh, James Q. Pham, Zhongying Z. Cui, David L. Dill, Harley H. McAdams, Lucy Shapiro

**Affiliations:** 1 Department of Developmental Biology, Stanford University School of Medicine, Stanford, California, United States of America; 2 Department of Computer Science, Stanford University, Stanford, California, United States of America; 3 Department of Electrical Engineering, Stanford University, Stanford, California, United States of America; Universidad de Sevilla, Spain

## Abstract

Each *Caulobacter* cell cycle involves differentiation and an asymmetric cell division driven by a cyclical regulatory circuit comprised of four transcription factors (TFs) and a DNA methyltransferase. Using a modified global 5′ RACE protocol, we globally mapped transcription start sites (TSSs) at base-pair resolution, measured their transcription levels at multiple times in the cell cycle, and identified their transcription factor binding sites. Out of 2726 TSSs, 586 were shown to be cell cycle-regulated and we identified 529 binding sites for the cell cycle master regulators. Twenty-three percent of the cell cycle-regulated promoters were found to be under the combinatorial control of two or more of the global regulators. Previously unknown features of the core cell cycle circuit were identified, including 107 antisense TSSs which exhibit cell cycle-control, and 241 genes with multiple TSSs whose transcription levels often exhibited different cell cycle timing. Cumulatively, this study uncovered novel new layers of transcriptional regulation mediating the bacterial cell cycle.

## Author Summary

The generation of diverse cell types occurs through two fundamental processes; asymmetric cell division and cell differentiation. Cells progress through these developmental changes guided by complex and layered genetic programs that lead to differential expression of the genome. To explore how a genetic program directs cell cycle progression, we examined the global activity of promoters at distinct stages of the cell cycle of the bacterium *Caulobacter crescentus*, that undergoes cellular differentiation and divides asymmetrically at each cell division. We found that approximately 21% of transcription start sites are cell cycle-regulated, driving the transcription of both mRNAs and non-coding and antisense RNAs. In addition, 102 cell cycle-regulated genes are transcribed from multiple promoters, allowing multiple regulatory inputs to control the logic of gene activation. We found combinatorial control by the five master transcription regulators that provide the core regulation for the genetic circuitry controlling the cell cycle. Much of this combinatorial control appears to be directed at refinement of temporal expression of various genes over the cell cycle, and at tighter control of asymmetric gene expression between the swarmer and stalked daughter cells.

## Introduction

**Figure 1:**
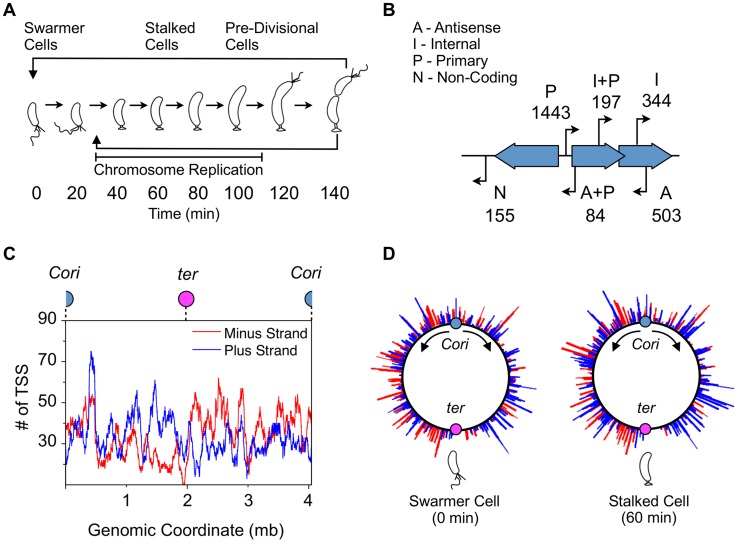
Global identification and organization of TSSs. (A) The *Caulobacter* asymmetric cell division cycle yields two distinct cell types: a motile swarmer cell and a sessile stalked cell. Stages of the *Caulobacter* cell cycle are shown in 20 min intervals, beginning with the swarmer cell at 0 min. Chromosome replication takes place between approximately 30 min and 110 min. TAP treated 5′ RACE sequencing libraries were prepared for each of the 8 time points shown. (B) Categories assigned to 2726 TSS identified based on their genomic context, plus the number of TSS in each category. TSS initiated upstream of CDSs are denoted as primary (P); those initiated in intergenic regions or upstream of annotated RNAs including small non-coding RNAs, ribosomal RNAs, and tRNAs are denoted as non-coding (N); those initiated from within coding sequences are denoted as internal (I), and those initiated within coding sequences but in the opposite direction are denoted as antisense (A). Some TSS fit the criteria for more than one category: primary and antisense (A+P, 84) and between primary and internal (I+P, 197). Many CDSs have multiple TSSs of different categories. (See Methods for specific definition of the categories.) (C) Distribution of TSSs on the genome, plotted as the number of TSSs within a 100kb sliding window. Plus strand: blue; minus strand: red. Blue dot: *Caulobacter* origin of replication (*Cori*). Purple dot: terminus (*ter*). (D) Distribution of TSS levels along the *Caulobacter* chromosome in swarmer (0 min) and stalked cells (60 min). Normalized number of sequencing reads (log scale) of cell cycle-regulated TSSs are shown. Blue: plus strand. Red: minus strand.

**Figure 2:**
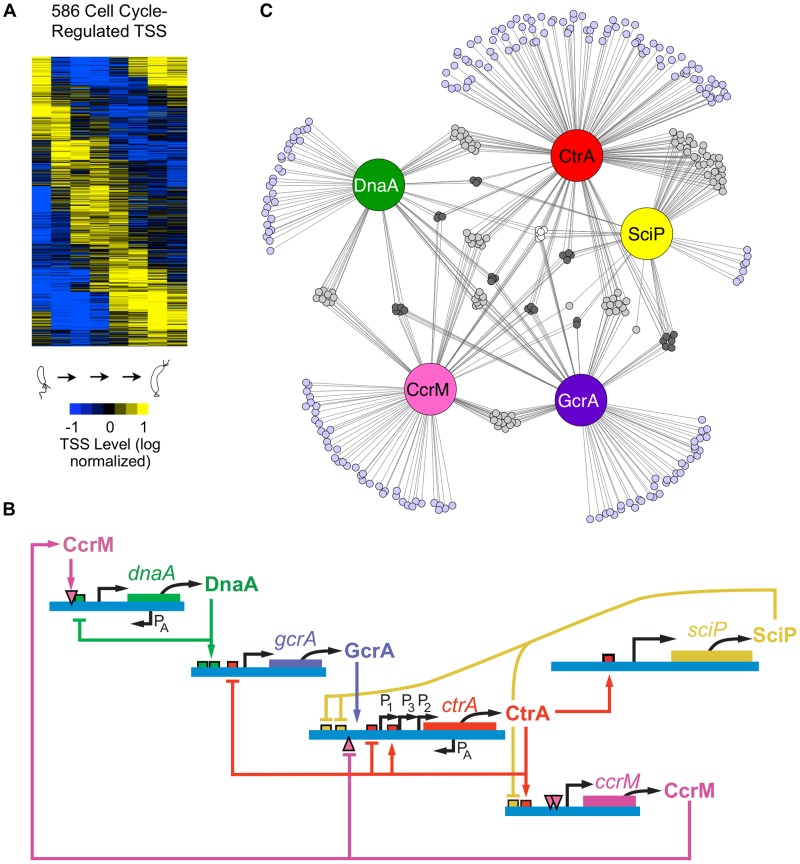
Cell cycle-regulated transcription by combinatorial control of master regulators. (A) Heat map of transcriptional profiles of the 586 cell cycle-regulated TSSs. Fifteen clusters were generated using the *k-means* algorithm and ordered according to their maximum time of activation as a function of the cell cycle. Columns correspond to the 8 time points during cell cycle progression shown in Fig. 1A and each row reflects the expression pattern of a single TSS. The TSS expression values are log_2_ transformed and normalized such that mean  = 0, max  = 1, and min  = −1. (B) The core regulatory circuit that drives the *Caulobacter* cell cycle. DnaA is required to initiate DNA replication, and it also acts as a transcription factor. The circuit contains transcription factors GcrA, CtrA, and SciP, as well as the DNA methyltransferase CcrM [2]. This core circuit drives cell cycle progression by orderly activation of the expression of 334 cell cycle-regulated TSSs. P_A_ indicates antisense TSSs within *dnaA* and *ctrA*. (C) Network of master regulators (DnaA, CtrA, SciP, and CcrM binding motifs or a>3 fold enrichment of GcrA Chip-seq signal [26]) in promoter regions of 334 cell cycle-regulated TSSs. Each promoter is represented by a small circle and each line represents interaction by a corresponding regulatory factors in a large colored circle. Promoters with ≥4 master regulators are white, 3 master regulators are dark grey, 2 master regulators are light grey, and 1 master regulator are light blue.

The regulation of timing and ordered progression of cell cycle events is central to the survival of any organism and is one of the fundamental processes of life. The gram-negative α-proteobacterium *Caulobacter crescentus* (*Caulobacter*, hereafter) is an important model organism for the study of the regulation of cell cycle progression and asymmetric cell division, shown in Fig. 1A [1]–[3]. A hallmark of *Caulobacter* asymmetric cell division is that the daughter stalked cell immediately initiates DNA replication and the daughter swarmer cell has a period of motility before differentiating into a stalked cell and initiating chromosome replication. Control of cell cycle progression and asymmetric division occurs through coordinate regulation of transcription, protein phosphorylation, DNA methylation, protein localization, and protein degradation [1], [4]–[6]. A cyclical genetic circuit, comprised of five master regulator proteins, including DnaA, GcrA, CtrA, and SciP, and the DNA methyltransferase CcrM, drives the cell cycle [2], [4] (see Fig. 2B). The circuit regulates the transcription of more than 200 genes controlling sequential polar differentiation events including flagella biosynthesis, pili biosynthesis, chemotaxis complex formation, DNA-replication, and cell division [3], [7]–[12]. However, the mechanism of cell cycle control for only a subset of these has been described. To decipher the regulatory landscape that guides the cell cycle we need to identify transcription start sites (TSSs), measure their cell cycle stage-specific levels, and define the regulatory motifs within each cell cycle-regulated promoter.

Here, using a detailed map of the coding and non-coding features in the genome based upon ribosome profiling [13], we applied global 5′ RACE to map approximately 75 percent of the *Caulobacter* TSSs at single base-pair resolution and to measure the abundance of RNAs carrying a 5′ tri-phosphate (5′ PPP) group. This was done at multiple time points during the cell cycle to determine the timing of activation of TSSs. We also identified binding sites of the key cell cycle regulatory transcription factors directly upstream of the TSSs. When multiple TFs were predicted to bind within these TSS-proximate regions, we were able to provide an initial estimate of the combinational control logic. For example, the core cell cycle circuit regulators DnaA and GcrA often regulate gene expression in combination with other transcription factors. For genes controlled by the cell cycle circuit regulator CtrA, the location and presence of full palindromic or half CtrA binding motifs, and co-appearance of SciP binding motifs, dictates the cell cycle timing of their transcriptional regulation. We discovered that 107 antisense TSSs positioned within Coding DNA Sequences (CDSs) are temporally regulated and identified 241 genes transcribed from multiple promoters whose activation is independently controlled, yielding different timing of TSS activation. Furthermore, we found internal promoters in operons that were independently regulated to alter the expression profiles of encoded genes. Cumulatively, these observations suggest that the regulation of *Caulobacter* TSS levels during the cell cycle is much more complex than previously reported and this dataset provides a powerful resource for the elucidation of the cell cycle regulatory circuit.

## Results

### Global identification of TSSs

We used a global 5′ RACE (rapid amplification of cDNA ends) method in combination with Illumina high-throughput sequencing to obtain a single-nucleotide resolution map of TSSs and their cell cycle-dependent activation level. Isolated swarmer cells (0 minutes) were grown in M2G minimal media for 140 minutes until cell division (Fig. 1A). We collected cell samples at 8 time points during the cell cycle and carried out total RNA extraction to prepare an Illumina high-throughput sequencing library for each time sample (S7 Fig. and Materials and Methods).

At each TSS the 5′ nucleotide contains a 5′ PPP group, whereas products of nuclease cleavage yield either a 5′ mono-phosphate (5′ P) group or a 5′ hydroxyl (5′ OH) group. As many processed RNAs such as mature ribosomal and transfer RNA (rRNA, tRNA) have a 5′ P, we prepared two additional libraries from unsynchronized culture in mid-exponential growth in minimal media to selectively distinguish between RNA segments with 5′ PPP ends and 5′ P ends. In one of these two libraries, the RNA was treated with tobacco acid pyrophosphatase (denoted +TAP) to hydrolyze the 5′ PPP to 5′ P. The other library (denoted -TAP) was prepared without TAP treatment (S7 Fig.). Libraries were ligated with a 5′ sequencing adapter followed by reverse transcription using a random-hexamer primer conjugated with a second Illumina sequencing adapter (S7 Fig.). Since T4 RNA ligase reacts only with a 5′ P, removal of the pyrophosphate group allows for the ligation of the 5′ sequencing adapter [14].

About 180 million 38 bp reads were obtained from the ten sequencing libraries (8 TAP treated cell cycle time point libraries and the +TAP and -TAP libraries from an unsynchronized mid-log phase culture). The reads were aligned onto the *Caulobacter* NA1000 genome DNA sequence NC_011916 [15] using Bowtie 0.12.7 software [16]. Only non-rRNA reads that mapped to a unique genomic location without mismatches were used for our analysis. Sequencing reads for all libraries were normalized to the total number of non-rRNA reads in each library.

To identify TSSs, we used 34 biochemically-characterized TSSs as a positive control (S2 Dataset) and 24 tRNA 5′ P-sites as a negative control (*n* = 24) (S1 Fig.). We compared the natural log of the ratio (*θ*) between the number of sequencing reads obtained at the 5′ ends of positive and negative controls in +TAP/-TAP libraries. The *θ*'s obtained for positive and negative controls fall into two separate normal distributions with slight overlap (Welch two sample *t*-test, df  = 41.2, *p*-value  = 1.3 e−11, S1 Fig.). To minimize false-positive TSSs, we set the threshold value at *θ*>0.26 which corresponds to approximately two standard deviations (*α* = 2.3%) above the sample mean of the negative control. We set the minimum read threshold in the +TAP asynchronous library to be 25. Using RNA-seq data from [13] we selected only TSSs that had more than a 35% increase in the downstream RNA-seq coverage (S1 Dataset). In total, this procedure identified 2,726 *Caulobacter* TSSs (S1 Dataset). The parameters we set for TSS identification allowed us to identify the TSSs for approximately 75% of genes and operons, in keeping with similar estimates of the percentage of TSSs reported for *Listeria monocytogenes* [17] and *Escherichia coli* [18]. Two factors are likely to contribute to the null identification of TSSs: strict parameter cutoff based on TAP-enrichment (S1 Fig.) and 5′ RACE dependence on a 5′ ligation, which is inefficient for RNAs that are highly structured at the 5′ region [19]. To verify our approach, we tested 36 of the TSSs identified by 5′ global RACE using *β*-galactosidase reporter assays and found that all 36 exhibited significant expression activity (S3 Dataset).

Canonical bacterial promoters contain binding sites for σ factors upstream of the TSS. A motif search of 50 bp upstream of the identified TSSs revealed a−35 (TTG) and −10 (A/T) binding site (*n* = 1,666) consistent with σ^73^ (RpoD), the most abundant housekeeping sigma factor in *Caulobacter* [20] (S2 Fig., S4 Dataset). The 5′ nucleotide of 93% (1,542/1,666) of the identified RpoD binding motifs are positioned between −34 to −37 bp upstream of the TSSs.

Based on recent functional re-annotations of the *Caulobacter* genome (CP001340) using ribosome profiling and computational analysis [13], [21], we categorize TSSs into four categories with overlap (Fig. 1B). TSSs located upstream of CDS are denoted as primary (P, 1443); those located in intergenic regions or upstream of annotated RNAs are denoted as non-coding (N, 155); those initiated from within coding sequences and transcribed in the same direction are denoted as internal (I, 344), and those transcribed in the opposite direction of the CDS are denoted as antisense (A, 503). There is overlap between primary and antisense TSSs (A+P, 84) and between primary and internal TSSs (I+P, 197). Thirty-three of the 93 previously characterized non-disruptable intergenic gaps in the *Caulobacter* genome [22] were found to contain a TSS within the non-disruptable gap, suggesting these TSSs may play a role in cell viability.

We also observed a slight directional bias in the number of TSSs encoded in the same direction as DNA replisome movement (*n* = 1544 co-directional, *n* = 1182 opposing) (Fig. 1C) to minimize collisions between DNA polymerase and RNA polymerase during chromosome replication [23]. Comparison of global TSS levels of the swarmer and stalked cell stage of the cell cycle revealed differences in the pattern of global site-specific TSS levels (Fig. 1D). We analyzed the direction of cell cycle-regulated TSSs peaking in the swarmer cell and find no significant directional bias (n = 21 co-directional, n = 19 opposing). As the swarmer cell does not actively replicate its chromosome, it is likely that these promoters have no selective pressure to be encoded co-directional with DNA replication.

### Cell cycle-regulated TSSs and enriched motifs

To distinguish cell cycle-regulated TSSs from constitutively active TSSs, we implemented a modified Fourier Transform algorithm, similar to [24], including both minimum sequence read and expression fold-change cutoffs on the corresponding normalized time-course sequencing data of the 2,726 identified TSSs. Using this approach, we identified cell cycle-regulated TSS levels for 586 TSSs (Fig. 2A). In general, the cell cycle TSS levels measured by 5′ global RACE yield similar timing as the steady state mRNA levels as determined previously by microarrays [25] (S3 Fig.).

To improve upon lower resolution and coverage studies of transcription factor binding motifs [25], we used our base pair resolution TSSs and a new genome annotation [13], [21] to search in DNA segments upstream of cell cycle-regulated TSSs for binding motifs of the core cell cycle circuit (Fig. 2B): CtrA (Fig 2C, S4 Fig., S5–S7 Dataset), SciP (Fig. 2C, S4 Fig., S8–S9 Dataset), DnaA (Fig. 2C, S4 Fig., S10 Dataset) and the CcrM DNA methyltransferase (Fig. 2C, S4 Fig., S11 Dataset). To identify TSSs regulated by the GcrA transcription factor, we searched upstream of TSSs for DNA segments enriched in GcrA ChIP-seq signal (S12 Dataset) [26]. About 57% of cell cycle-regulated TSSs had upstream binding motifs for one or more of the four transcription factors or CcrM DNA methlytransferse in the core cell cycle regulatory circuit (Fig. 2C, S4 Fig., S5–S12 Dataset). We identified 199 cell cycle regulated TSSs with a single upstream regulatory binding motif for one of the known master regulators (DnaA, 29; GcrA, 34; CtrA, 89; SciP, 7; CcrM, 40). In addition, we found another 135 TSSs that have multiple master regulatory factor binding sites, suggesting they are under combinatorial control (S13 Dataset). The TSSs that are preceded by multiple regulatory motifs are enriched for genes encoding critical cell cycle proteins, including the genes of the core regulatory circuit itself.

We also identified binding motifs for sigma factors SigT (S5 Fig., S14 Dataset) and RpoN (S5 Fig., S15 Dataset). The TSSs with binding motifs for SigT, a cell cycle-regulated ECF sigma factor [11] that is also induced under stress [27], exhibited peak levels during the swarmer to stalked cell transition coincident with the expression pattern of *sigT* (S5 Fig.). The previously identified *Caulobacter* SigT binding motif (GGAAC-N16-CGTT, e-value  = 1.9 e−39, *n* = 26) [25], [27] is located at the −35 bp region relative to the TSS (S5 Fig.). RpoN, a sigma factor induced upon nitrogen limitation and required for flagella gene expression in *Caulobacter* [28], [29], controls two classes of genes in both the swarmer to stalk transition and in flagellar genes. The previously identified RpoN binding motif (GGCNC-N4-CTTGC, e-value  = 1.5 e−27, *n* = 33) [25], [30] is located between −35 bp and −25 bp relative to the TSS (S5 Fig.). The RpoD, SigT, and RpoN binding motifs together account for 63% (1725/2726) of the observed upstream TSSs regions. The remaining TSSs are likely activated by the additional 13 known sigma factors encoded in the *Caulobacter* genome.

**Figure 3:**
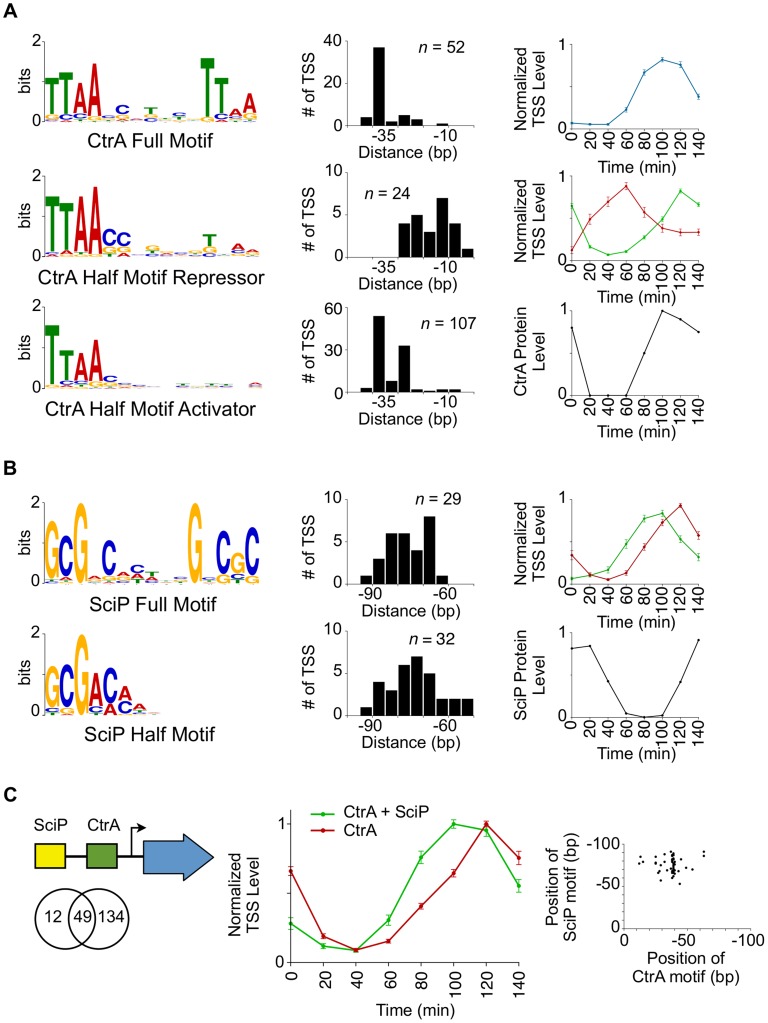
Coordinated control of CtrA regulated transcription by SciP. (A) CtrA full motif TTAA-N7-TTAA (n = 52, *e*-value  = 1.2 e−49) (top left). Histogram to the right represents the distances from which the 5′ nucleotide of the CtrA full motif is positioned relative to the TSS. Normalized TSS levels for the full CtrA motif as a function of the cell cycle (top right). Normalized TSS levels (*y*-axis) indicates the fraction of reads relative to the maximum obtained during the cell cycle, and error bars represent standard error. Left middle and left bottom graphs show the CtrA half motif, TTAA, enriched in two separate groups that reflect their position relative to the TSS (shown to the right). Normalized TSS activities are shown on the right middle panel in red (CtrA half motif repressor, n = 24, *e-*value  = 5.2 e−8) and green (CtrA half motif activator, n = 107, *e*-value  = 4.2 e−141). CtrA protein levels as a function of the cell cycle, normalized from western blots [34] (max  = 1, min  = 0), are shown in the bottom right panel. (B) SciP full motif GCGNC-N5-GNCGC (n = 29, *e*-value  = 3.5 e−19, top left) and half motif GCGNC (n = 32, *e-*value  = 3.4 e−6, bottom left) are enriched in two separate groups. Histograms of the SciP full motif (red) and half motif (green) are shown relative to the TSS. The normalized TSS activities as a function of the cell cycle are shown on the right in red (full motif) and green (half motif). Shown in the bottom right, SciP protein levels are normalized from western blots [8] (max  = 1, min  = 0). (C) Representation of relative positions of SciP and CtrA binding motifs (Left); Venn diagram showing the number of TSS with an enriched upstream CtrA motif, SciP motif, or both. Middle panel shows normalized TSS levels as a function of the cell cycle showing CtrA and SciP binding motifs (green) or CtrA only (red). Only TSSs activated by CtrA are included. Error bars are the standard error. Scatter plot of the coincident position of SciP and CtrA binding motifs (bottom right).

The CtrA response regulator is a master transcriptional regulator of the *Caulobacter* cell cycle that was shown to directly control 95 cell cycle-regulated genes using ChIP-chip [11], [12]. We identified 183 cell cycle-regulated TSSs with an upstream CtrA binding motif (Fig. 3A) that were also enriched in CtrA ChIP-seq data [31] (S5–S7 Dataset). Among these are promoter regions of the cell cycle master regulators *sciP, ccrM,* and *ctrA*, a regulator of CtrA degradation *rcdA*, cell division proteins *ftsK*, *ftsQ*, and *mipZ*, the response regulator *divK*, flagellar genes, and 6 non-coding RNAs (S5–S7 Dataset). Surprisingly, we observed two classes of CtrA binding motifs, a full palindromic TTAA-N7-TTAA (Fig. 3A) and a half motif TTAA (Fig. 3A). Based on hierarchical clustering of the cell-cycle profiles we identified 3 groups of CtrA binding motifs (CtrA full, CtrA half repressor, and CtrA half activator). Expression of genes controlled by CtrA full (n = 52, S5 Dataset) mirrored CtrA protein levels in the predivisional cell stage (60–120 min) (Fig. 3A). The 5′ nucleotide of these motifs is positioned near the −35 region, consistent with CtrA activity as a transcriptional activator in the predivisional cell. Conversely, CtrA half repressor containing promoters (n = 24, S6 Dataset) exhibited an anti-correlated temporal pattern of TSS activation with the CtrA protein levels (Fig. 3A). These half sites were positioned over the −10 site, consistent with CtrA functioning as a transcription repressor, similar to the observed repression of *ctrA* P1 by CtrA [32] (Fig. 2B). Fifty eight percent (14/24) of promoters with a CtrA half repressor motif also contain a DnaA binding motif or a GcrA binding site. CtrA half site-containing promoters that function as activators (n = 107, S7 Dataset) correlated with CtrA protein levels throughout the cell cycle, with transcription activity occurring in both the swarmer and predivisional cell stages. These CtrA half activator binding sites are also near the −35 region, consistent with transcriptional activation (Fig. 3A). Indeed, gene expression profiling studies using microarrays from strains with altered CtrA activity [28] show good agreement with CtrA full and CtrA half activator in transcription activation and CtrA half repressor in transcriptional repression (S5–S7 Dataset). These data show that the activity and timing of transcription is controlled by the precise position of CtrA DNA binding upstream of the TSS. Additionally, two transcription factors (MucR 1/2) that act to regulate the S-G1 phase transition were reported to bind to CtrA target promoters. We found that 76% of cell cycle-regulated promoters under MucR 1/2 ChIP-seq peaks, as determined by the Fumeaux *et al.* [33], contains a CtrA binding motif, and 10% of the cell cycle-regulated promoters under MucR 1/2 ChIP-seq peaks contain a SciP binding motif. Those CtrA regulated promoters under MucR 1/2 peaks were more highly repressed in the stalk/early-predivisional cells than CtrA-regulated promoters without MucR 1/2 (S10 Fig.). This is consistent with the proposal by Fumeaux *et al.* [33] that MucR 1/2 specifically represses CtrA activated genes in the S phase while SciP specifically represses CtrA activated genes in the swarmer cell.

There are a total of 61 TSSs with a SciP binding motif [8] in the upstream promoter region, and among these are the promoter regions of *ctrA*, the DNA methyltransferase *ccrM*, and polar development protein *podJ*. Previously, the promoter regions of 30 genes were identified as targets of SciP by expression arrays [7], 15 of which were shown to be direct by ChIP-PCR [8]. Seven of the 15 promoter regions shown to directly bind SciP, were identified by the TSS motif search. As with the CtrA binding motifs, we found that the SciP motif falls into two categories: a palindromic motif GCGNC-N5-GNCGC and a half motif GCGAC (Fig. 3B) that was identified previously (reverse complement in [8]). TSSs with the palindromic motifs exhibited peak levels at 120 min (*n* = 29, S8 Dataset) and those with half SciP motifs exhibited levels peaking at 100 min (*n* = 32, S9 Dataset) (Fig. 3B). Both groups exhibited an anti-correlated cell cycle profile with SciP protein levels indicating SciP acts as a repressor, in agreement with previous reports [7], [8]. To confirm this role as a repressor, we showed that the mutation of the SciP site in three CtrA activated promoters leads to an increase in the promoter activity (S9 Fig.). The half motif is associated with early TSS repression and the full motif with repression later in the cell cycle (Fig. 3B). SciP binding motifs are predominantly found between −60 bp and −90 bp relative to the TSS (Fig. 3B–C) and 80% (49/61) of TSSs with a SciP binding motif also have a CtrA binding motif (Fig. 3C). On average the SciP ChIP-seq signal peak occurs upstream of the CtrA ChIP-seq signal in agreement with the upstream position of SciP binding motifs relative to CtrA (S8 Fig.). When SciP sites are combined with CtrA sites, SciP represses genes in the late predivisional cell and the swarmer cell (Fig. 3C) where SciP protein levels peak (Fig. 3B).

The DnaA protein directs the initiation of chromosome replication in addition to functioning as a transcription factor [10]. We identified DnaA binding motifs in 77 promoter regions of cell cycle regulated TSSs (S10 Dataset). DnaA regulates transcription of GcrA, FtsZ, PodJ, and components of the replisome and nucleotide biosynthesis proteins [10]. The DnaA binding motif occurs as the sole master regulator site in 29 promoter regions, while it is commonly accompanied at promoter regions by 25 CtrA, 19 CcrM, and 19 GcrA sites (Fig. 2C, S4 Fig.).

The GcrA protein is a master transcription factor that is activated by DnaA (Fig. 2B) [34] and whose protein levels are anti-correlated with CtrA [9]. We searched for enrichment of the GcrA signal in promoter regions of cell cycle regulated TSSs in the ChIP-seq dataset reported by [26] and found GcrA binding sites in promoters of CtrA P1, *podJ*, and *mipZ* in addition to 91 other promoter region (S12 Dataset). GcrA binding occurs as the sole master regulator site in 34 promoter regions, while it is accompanied by 30 CtrA, 29 CcrM, and 19 DnaA binding motifs (Fig. 2C, S4 Fig.).

**Figure 4:**
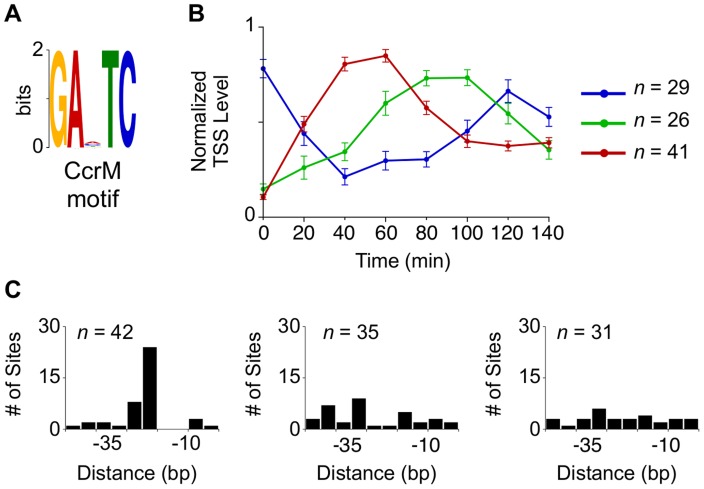
Cell cycle-regulated TSSs with upstream CcrM methylation sites. (A, B) CcrM methylation motif GANTC (n = 108, *e*-value  = 2.0 e−56) enriched in the promoter regions correspond to three separate groups of TSSs generated by hierarchical clustering with average cell cycle profiles shown in (blue, red, and green). (C) Histogram of the distances in which the CcrM GANTC methylation motif is found relative to the TSS.

Hemi-methylated GANTC sites in the *Caulobacter* chromosome are recognized by the CcrM DNA methyltransferase yielding 6-methyl adenines [35], [36]. The transcription of CtrA and DnaA is affected by the cell cycle-dependent methylation state of their promoters, a link that helps synchronize the progression of the core cell cycle regulatory circuit with the progress of DNA replication [3], [37], [38]. We identified a total of 96 TSSs with GANTC sites located within 50 bp upstream of the TSS that were activated at specific stages in the cell cycle (Fig. 4A–B, S11 Dataset). Eleven of the 96 TSSs contain more than one upstream GANTC site yielding a total of 108 GANTC sites within 50 bp upstream of cell cycle regulated TSSs. These cell cycle-regulated TSSs fell into three distinct temporal clusters. For the TSSs with the GANTC site positioned between −10 and −35 of the promoter region, the time of TSS activation occured between 40 and 60 minutes (Fig. 4B–C red). If the GANTC motif is positioned outside this region the TSS levels is lowest between 40 and 60 minutes (Fig. 4B–C blue). A third cluster of cell cycle-regulated TSSs that have GANTC sites equally distributed within 50 bp upstream of the TSS exhibits maximal levels between 80 to 100 minutes (Fig. 4C green). Fifty-eight percent of all TSSs with upstream GANTC motifs contained other master regulator binding motifs (Fig. 2C, S4 Fig.).

### Dynamic expression of antisense TSSs

Based on this TSS study and a recent RNA-seq study [13], we identified 587 (503 A, 84 A+P) antisense transcripts in the *Caulobacter* genome. Only eight antisense transcripts for genes encoding transposases have been reported previously [39]. We found an additional 179 putative antisense TSSs that have RNA-seq coverage [13] below our mapping threshold (S16 Dataset). Despite the low RNA-seq coverage, 7 (out of 7 assayed) antisense TSS with low RNA-seq coverage had significant β-galactosidase activity when 75 bp of the promoter were inserted in front of the β-galactosidase gene (S3 Dataset), suggesting they are indeed antisense TSSs. 583/3,885 (∼16%) of *Caulobacter* CDSs have at least one antisense TSS; as compared to *Helicobacter pylori* (27%) [40], *Escherichia coli* (20%) [41], [42], and *Mycoplasma pneumoniae* (12%) [43]. Seventy-four antisense TSSs reside within essential genes including those that encode DnaA, CtrA, an RNA polymerase beta chain, and MreB.

**Figure 5:**
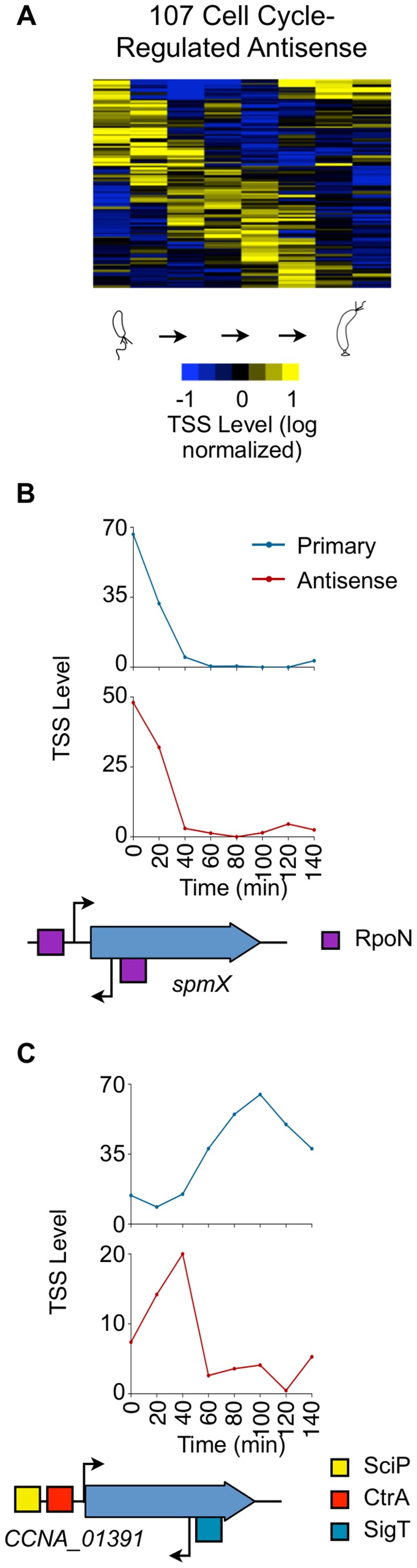
Cell cycle-regulated antisense TSSs. (A) Heat map of transcriptional profiles of the 82 cell cycle-regulated antisense TSSs (A or A+P) *k*-means clustered and ordered by time of activation. Columns correspond to time points in the cell cycle (0–140 min) in 20 min intervals, and each row denotes a single TSS. All TSS expression values are log_2_ transformed and normalized such that mean  = 0, max  = 1, and min  = −1. (B, C) Cell-cycle transcriptional profiles of primary (blue) and antisense (red) TSS of *spmX* and *CCNA_01391*, respectively. Time (min) is shown on the *x*-axis. Normalized 5′ RACE sequencing reads shown on *y*-axis. Locations of TSS as well as SciP (yellow), RpoN (purple), and SigT (blue) binding motifs with respect to the CDS are represented below the *x*-axis. *CCNA_01391* has a SciP binding motif (GCGNC) and a CtrA binding motif (TTAT-N7-TCAA) at −79 and −32 of its leaderless primary TSS respectively. There is a SigT binding motif (GGAAC-N16-TGCT) at −34 of the *CCNA_01391* antisense TSS. The gene *spmX* has a RpoN binding motif (GGCNC-N4-CTTGC) at −26 bp relative of its primary TSS and another putative RpoN binding motif (CGCAC-N4-CTTGC) at −24 relative to its antisense TSS.

Of the 766 (587+179) antisense TSSs in the *Caulobacter* genome, 107 are cell cycle regulated (Fig. 5A). Of these 107, 42 are within genes that are constitutively expressed and 13 are within genes that are cell cycle controlled. We observed for the *spmX* gene, that promotes the localization and activation of the DivJ histidine kinase [44], the timing of antisense transcription is correlated with the timing of sense transcription (Fig. 5B). Perhaps the antisense transcript stabilizes the *spmX* mRNA, as reported for the *gadX* mRNA by the antisense GadY transcript in *E. coli* [45]. In 12 of the 13 cell cycle-regulated antisense TSSs residing within with a cell cycle controlled gene (S17 Dataset), the levels of the antisense TSS and the corresponding cell cycle-regulated primary TSS peak at different times over the course of the cell cycle, as shown for *CCNA_01391* (Fig. 5C). The antisense TSS with a SigT binding site in its promoter is induced at the swarmer to stalked cell transition. Upon the decrease in levels of the antisense TSS, we observe an increase in the levels of the primary TSS (Fig. 5C). The coordinated transcriptional patterns of these genes and their antisense TSSs, suggest that the antisense RNA may control gene expression.

### Cell cycle-regulated intergenic non-coding TSSs

**Figure 6:**
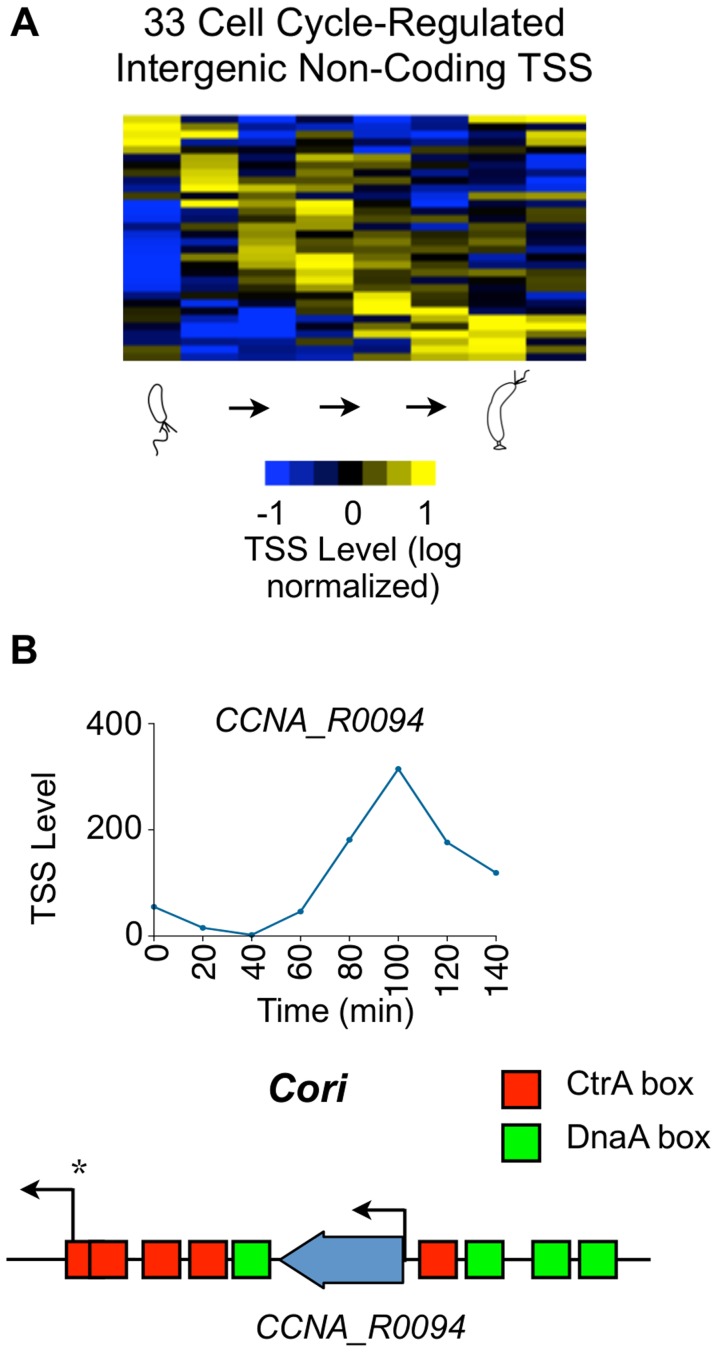
Cell cycle-regulated intergenic non-coding TSSs. (A) Heat map of transcriptional profiles of the 33 cell cycle-regulated intergenic non-coding TSS clustered (using *k*-means clustering algorithm) and ordered by time of activation. Columns correspond to time points as a synchronized culture progresses from swarmer cell (0 min) to cell division (140 mins) in 20 min intervals, and each row denotes a single TSS. All TSS expression values are log_2_ transformed and normalized such that mean  = 0, max  = 1, and min  = −1. (B) Cell cycle profile of the TSS of ncRNA gene *CCNA_R0094* in terms of the normalized 5′ RACE reads vs time. *CCNA_R0094* is transcribed from within the chromosomal origin region (*Cori*). Locations of the CtrA (red) and DnaA (green) binding boxes as reported by [82] are shown. Transcription from the promoter denoted by * is essential for chromosome replication [83].

Bacterial intergenic small non-coding RNAs (ncRNAs) have been shown to enable cells to adapt to environmental and physiological challenges [46]. We have separately reported 199 ncRNAs in the *Caulobacter* genome [13], including four new ncRNAs that are encoded in nondisruptable regions of the genome [22]. We identified 155 TSSs within intergenic non-coding regions (category N, Fig. 1B) using 5′ global RACE in minimal medium; these included 50 TSSs for tRNA or rRNA genes. In 46 of these 155 intergenic TSSs, the TSS matches the 5′ nucleotide in the ncRNA identified by RNA-seq [13] (S18 Dataset). While only 5 *Caulobacter* ncRNAs were previously observed to be cell cycle-regulated [39], [47], we identified 33 cell cycle-regulated non-coding TSSs activated in different phases of the cell cycle (Fig. 6A, S1 Dataset). One of these cell cycle-regulated TSS drives a ncRNA of 182 nt in length (CCNA_R0094) transcribed from within the chromosomal origin of replication (Fig. 6B) that appears to be essential [22].

### Cell cycle regulation of CDSs with multiple promoters

**Figure 7:**
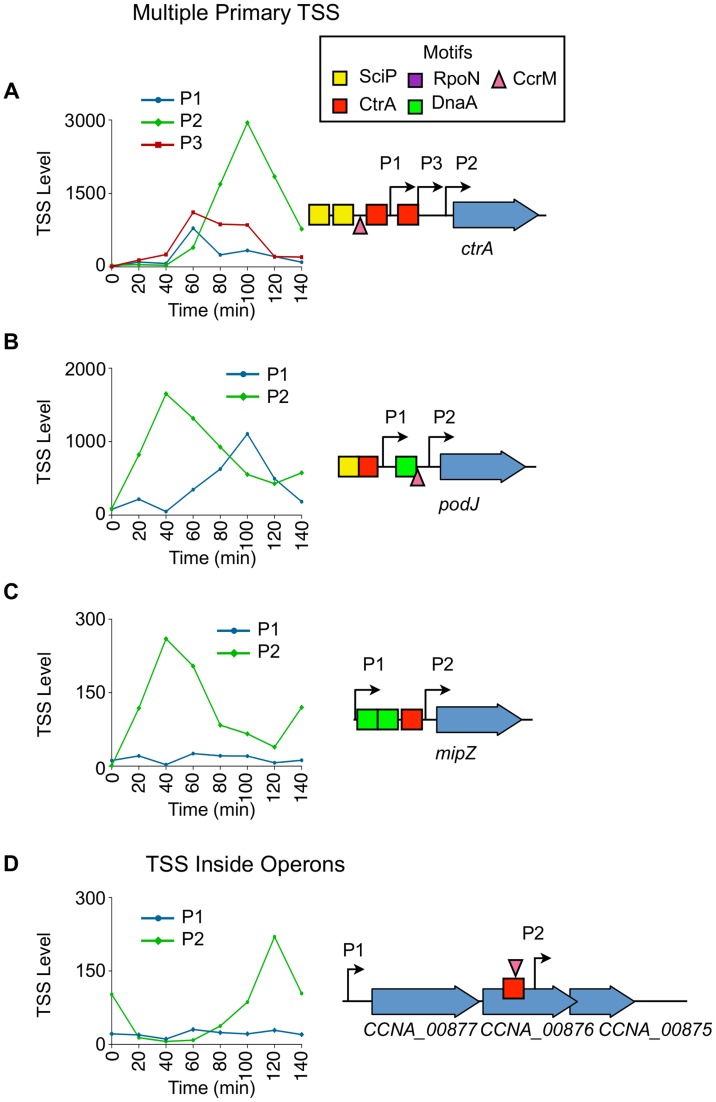
Cell cycle-regulation of genes with multiple upstream TSSs. (A) Cell-cycle profile of the 3 cell cycle-regulated TSSs (P1, P2, P3) upstream of *ctrA.* Time (min) is shown on the x-axis. Normalized 5′ RACE reads shown on y-axis. P1 in blue, P2 in green, and P3 in red. Locations of P1, P2, and P3 with respect to the CDS is shown on the right. The CcrM methylation site GANTC (▾) is located at −29 of P1. The CtrA half motif repressor TTAA (red box) is located at −14 of P1, and the second CtrA binding motif TTAA-N7-TTAA is located at -39 of P2 and at −14 of P3. Two SciP half binding motifs GCGNC (yellow boxes) located −78 of P1 and −74 of P3. (B, C) Cell-cycle profiles of multiple TSS upstream of *podJ and mipZ*. P1 plotted in blue, and P2 plotted in green. Time is shown on the x-axis.; normalized 5′ RACE reads on the y-axis. The location of P1 and P2 with respected to the CDS is represented on the right. Both P1 and P2 of *podJ* are cell cycle-regulated. CcrM methylation sites GANTC (▾) located at −24 of *podJ* P2. P2 of *mipZ* is cell cycle-regulated. There is a CtrA full motif TTAA-N6-TTAA at −49 of *podJ* P1 and a CtrA half motif repressor TTAA at −10 of *mipZ* P2. A SciP binding motif GCGAC is located at −72 of *podJ* P1. DnaA binding motifs (green boxes) CTCCACA, ATCCACA, and GTCCACA at −82 of *podJ* P2 and −52 and −83 of *mipZ* P2 respectively. (D) Cell-cycle profiles of TSS upstream (P1) and inside (P2) the operon consisting of *CCNA_00877*, *CCNA_00876*, and *CCNA_00875*. Time (min) is shown on the x-axis. Normalized 5′ RACE reads shown on *y*-axis. The location of P1 and P2 with respected to the CDS is represented on the left. P2 is cell cycle-regulated, and there is a CcrM methylation site GANTC (▾) at −35 of P2. There is a CtrA binding motif (TTAC-N7-TTCA) at -39 of P2 inside *CCNA_00876*.

We identified a total of 241 CDSs with multiple upstream TSSs (S19 Dataset) that appear to be independently controlled. Fifty seven of these CDSs containing multiple promoters are essential for viability [22]. In *Caulobacter*, only 18 cell cycle-regulated genes, including *ctrA*, *dnaN*, *clpX*, and *rcdA*, have been shown previously to be transcribed from multiple cell cycle controlled promoters using either tiling microarrays, nuclease protection, or primer extension assays [25], [32], [48], [49]. We found that 102 CDSs (42% of those with multiple upstream TSSs) have at least one cell cycle-regulated TSS, and 25 CDSs, including *ctrA* (3 promoters, Fig. 7A) and *podJ* (2 promoters, Fig. 7B), have more than one cell cycle-regulated primary TSS that are independently regulated.

The *ctrA* gene was previously shown to be transcribed from two promoters (P1 and P2) where P1 is activated by GcrA after the *ctrA* locus is replicated and P1 becomes hemi-methylated (Fig. 2B) [9], [37]. The P1 promoter is thereafter repressed by CtrA which simultaneously strongly activates P2 [32]. We confirmed the previously reported temporal sequence of P1 and P2 activation, and identified an additional cell cycle-regulated promoter, P3, located between P1 and P2 (Fig. 7A). A CtrA half repressor binding motif TTAA is located −14 bp upstream of the P1 TSS, in addition to a SciP binding motif GCGAC located −78 bp upstream, and a CcrM methylation site GANTC (▾) located at −28 bp upstream of P1 (S6, S9, S11 Dataset). A full CtrA binding motif TTAA-N7-TTAA is located at −39 bp upstream of P2 and at −14 bp upstream of P3, and a half SciP binding site is located at −74 bp upstream of P3 (S5, S9 Dataset). Both SciP and CtrA have been shown previously to bind at both locations [8], [32]. The full CtrA motif likely functions to activate P2 and simultaneously repress P3 because the 5′ nucleotide of the CtrA full motif is at −14 bp upstream of P3 (repression) and at −39 bp upstream of P2 (activation).

PodJ is an essential protein that mediates polar organelle development by contributing to the synthesis of pili and the control of the polar localization of the PleC kinase/phosphatase [50], [51]. Two cell cycle-regulated primary TSSs (P1 and P2) are located 120 bp and 22 bp upstream, respectively, of *podJ* (Fig. 7B). The levels of P2, which contains a CcrM methylation site GANTC (▾) −24 bp upstream (S11 Dataset) and a DnaA binding motif (CTCCACA) (Hottes et al, 2005) at −82 bp upstream (S10 Dataset), peaks at 40 min into the cell cycle during the swarmer to stalked cell transition. P1 contains a CtrA full binding motif TTAA-N6-TTAA at −49 bp upstream (S5 Dataset) and a SciP half binding motif GCGAC at −73 bp upstream (S9 Dataset) with P1 levels peaking at 100 min into the cell cycle in the predivisional cell, contributing to a second wave of *podJ* transcription.

For 77 out of the 241 CDSs transcribed with multiple primary TSSs one promoter is constitutively expressed while the other is cell-cycle regulated. For example *mipZ* (Fig. 7C), which encodes the essential division plane positioning ATPase MipZ [52], has a constitutive P1 and a cell cycle-regulated P2 promoter containing a CtrA half repressor motif at −10 bp upstream of the TSS (Fig. 7C, S6 Dataset). Activation of the P2 promoter results in an increase in the transcription of MipZ at the same time as transcription of FtsZ, thereby coordinating the temporal control of MipZ regulation of FtsZ function [6].

### Dynamic transcription within operons

In many instances, the transcription of individual genes within the 848 operons is differentially regulated. There are 115 operons that have a cell cycle-regulated TSS upstream of the leading CDS. There are 52 operons (S20 Dataset) with internal cell cycle-regulated TSSs for downstream CDSs enabling independent cell cycle regulation of downstream operon genes. One example is the operon consisting of *CCNA_00875*, *CCNA_00876*, and *CCNA_00877*, where *CCNA_00875* encodes a 7,8-dihydro-8-oxoguanine-triphosphatase, *CCNA_00876* encodes a Flp/Fap pilin component protein, and *CCNA_00877* encodes a protein of unknown function (Fig. 7D). In this operon, the P1 start site is constitutively active, but the P2 start site has a putative CtrA full binding motif TTAC-N7-TTCA upstream of it (S5 Dataset) where the 5′ nucleotide of the motif resides at -39 bp and a CcrM methylation site GANTC (▾) is at −35 bp (S11 Dataset). The P2 TSS has a cell cycle-dependent profile suggesting cell cycle-regulated expression of just the downstream CDSs in the operon.

## Discussion

We used a global 5' RACE method to identify TSSs at single base pair resolution for approximately 75% of *Caulobacter* genes and identified their pattern of cell cycle regulation. Previous studies have identified *Caulobacter* cell cycle-regulated steady state mRNA levels using microarrays or RNA-seq [10], [11], [25], [53]. Here, we measured the abundance of 5′PPP ends corresponding to the relative activity of individual promoters at different time points during the cell cycle. We identified 586 cell cycle-regulated TSSs and assayed their time and level of activation as a function of cell cycle progression. This detailed map of TSSs and their temporal profiles of transcription activation have revealed new layers of gene regulation by the cell cycle control circuit.

### Antisense TSSs

Genome-wide assays have shown that antisense transcription occurs in many bacteria species [40], [41], [43]. Further, antisense transcripts have been shown to regulate genes involved in a wide variety of processes such as photosynthesis in *Synechocystis* PCC6803 [54], acid and SOS response in *E. coli* [45], [55], virulence in *Salmonella enterica* [56], and iron transport in *Vibrio anguillarum* [57]. The 107 cell cycle-regulated antisense TSSs suggest that antisense regulation is a significant element of cell cycle regulation. Supporting this, we found that 23 of the 107 cell cycle regulated antisense TSSs have binding sites in their promoter regions for the core cell cycle regulatory factors. Antisense TSSs are also found within the CDSs of essential cell cycle-regulated genes, including *dnaA*, *ctrA*, *spmX* and *mreB*. Most previously described mechanisms of antisense transcripts involve base-pairing with the corresponding sense mRNA to alter the RNA stability [45], [54], translation [58], or transcription termination [57]. Depending on the context, the antisense RNA has been observed to effect either down-regulation or up-regulation of genes. In *Caulobacter*, for 12 cell cycle regulated primary TSSs, the TSS levels is anti-correlated with the time of the time of activation of the antisense TSS (S17 Dataset), suggesting that antisense transcripts may down-regulate the levels of their target mRNAs. Additionally, the *Caulobacter* antisense TSSs show multiple cell cycle expression patterns, suggesting that this mechanism is active in regulation of gene expression during all stages of the cell cycle. Twenty four promoters upstream of the 107 cell cycle-regulated antisense TSSs contain master cell cycle regulator binding motifs allowing them to be controlled directly by these cell cycle regulating transcription factors. Antisense TSSs are also abundant in *Sinorhizobium meliloti* [59], another α-proteobacteria. However, as master regulator binding motifs residing within the protein coding sequences have generally not been included in global analyses of the α-proteobacterial cell cycle transcription control circuitry [25], [60], [61] and it is not known whether antisense TSSs controlled by the cell cycle master regulators are conserved within the α-proteobacteria.

### Noncoding TSSs

In *Caulobacter*, 199 intergenic ncRNAs have been identified in the genome including rRNAs and tRNAs [13], [39]. Of these, we identified 155 TSSs for ncRNAs (S1 Dataset) and for 33 of these the TSS levels are cell cycle-regulated (Fig. 6A). The functions of only two ncRNAs (non rRNA/tRNA/RNaseP/4.5S RNA) have been characterized: the tmRNA which rescues stalled ribosomes and alters the timing of replication initiation, and *crfA* which controls the carbon starvation response [47], [62], [63]. While ncRNAs perform many functions, a common ncRNA function in bacteria involves ncRNA base-pairing to mRNAs to regulate gene expression, sometimes mediated by the RNA chaperone Hfq [46], [64]. Four of the ncRNAs lie within non-disruptable intergenic gaps [22] suggesting that these ncRNAs may be essential for the regulation of *Caulobacter* cell cycle progression.

### Regulation by multiple promoters

Across the *Caulobacter* genome we identified 241 CDSs with multiple upstream TSSs (S19 Dataset) including 57 of essential [22] and 102 of cell cycle regulated genes. The genes encoding the CtrA global cell cycle regulator and the PodJ polar differentiation factor have multiple upstream promoters with different cell cycle timing that modulates the pattern of expression of the genes. In the case of *ctrA* transcription, GcrA activates the hemi-methylated P1 promoter to initiate *ctrA* transcription followed by a boost in CtrA production from the subsequent auto-regulated activation of the P2 promoter [9], [32] (Fig. 2B, Fig. 7A). Based on the identification of a third temporally controlled promoter, P3, whose temporal pattern of activation is similar to that of P1, we suggest that P1 and P3 accelerate initial production of CtrA. Both P1 and P3 have a CtrA binding site in the −10 region upstream of each TSS, which are then repressed by CtrA. We also know, as mentioned earlier, that the subsequent expression of SciP represses *ctrA* transcription. The net effect appears to be aimed at modulating the shape of the pulse of CtrA production to make it stronger, yet shorter in time.

In the case of *podJ*, the function of the additional promoters seems to be to extend of the duration of PodJ production over a longer interval of the cell cycle. In other cases, such as the cell division gene *mipZ*, a cell cycle-regulated promoter is activated at a specific time during the cell cycle, corresponding to the time in which the protein product is needed, but the gene is also transcribed at a low level from a constitutive promoter, presumably ensuring that a low level of MipZ is always present during the cell cycle (Fig. 7C).

We found that 209 operons contain internal TSSs, 55 of which are cell cycle-regulated (S20 Dataset). In some operons, downstream genes are activated at different times in the cell cycle. Internal TSSs that exhibit independent cell cycle-regulated expression have nearby upstream transcription factor binding motifs. Additionally, we report separately that some promoters internal to operons lead to production of alternative shortened forms of the encoded protein [13], presumably changing the protein's function. The spatial ordering of multiple upstream promoters, in conjunction with promoters internal to operons, could conceivably have a regulatory impact since each mRNA has a different 5′ UTR sequence that would enable differential post-transcriptional control. The exciting implications of the combinatorial promoter logic possible for genes and operons with multiple TSSs remain to be explored.

### Control of the CtrA regulon

The largest class of cell cycle-regulated TSSs are those that have one or more CtrA binding motifs in the promoter region (183 of the 586 of the cell cycle-regulated TSSs) (Fig. 2C, 3A, S4 Fig.). We observe two CtrA binding motifs, a full palindromic binding site and a half palindromic binding site (Fig. 3A). We find that the 5′position of the CtrA full palindromic motif relative to the TSS is most commonly at the −35 region corresponding to activation of transcription in the predivisional cell. Conversely, the 5′position of the CtrA half-palindromic motifs can either function as a repressor by binding over the −10, or an activator by binding over the −35 region. In predivisional cells, CtrA full-palindromic TSSs maximally activate at 80 to 100 min while those with CtrA half activator, do so later at the 120 min time point, likely due to the tighter binding affinity of the CtrA full site to CtrA∼P [65]. TSSs with CtrA half motifs are also active in the swarmer cell, while CtrA full motif TSSs are only active in the predivisional cell. We interpret this switch in TSS levels to be primarily due to overlapping control by SciP which inhibits CtrA activated promoters as reported by [7], [8]. About 80% (49/61) of TSSs containing an upstream SciP motif also have a CtrA motif (Fig. 2C, 3C, S4 Fig.). The SciP binding motifs are positioned between −60 and −90 upstream of the TSSs (Fig. 3B, S8 Fig.). SciP binds directly to DNA as shown in [8] and confirmed here by mutation of SciP sites. We have mutated both the half and full SciP sites in 3 CtrA activated promoters and in each case we observed an increase in promoter activity as measured by β-galactosidase activity, providing evidence that the SciP motif does indeed function to bind SciP and acts that bound SciP is a repressor of these promoters (S9 Fig.). In the presence of DNA containing both CtrA and SciP binding motifs, both CtrA and SciP become resistant to proteolysis [66]. Since direct interaction between CtrA and SciP has been demonstrated [7], [8], it seems likely that the combined DNA binding energy provides additional regulatory capacity. Interestingly, SciP and CtrA binding motifs are primarily positioned together with at least one motif present as a full palindromic binding site (91.8% of co-regulated promoters) and the co-positioning of SciP and CtrA half motifs occurs only in four co-regulated promoters. It is possible that SciP and CtrA require sufficient binding to DNA to form a stable complex that is not accomplished with weaker half sites.

A recent report by Fumeaux *et al.* [33] analyzed the top 50 SciP ChIP-seq peaks and found them to contain TTAACAT motifs, similar to the CtrA half binding motif. We performed motif searches of the SciP ChIP-seq peaks reported by Fumeaux *et al.* [33] using the CtrA half- and SciP half-binding motif presented in this paper, and found a total of 63 CtrA half motifs and 143 SciP half motifs with a P value less than 1^−3^. Both Fumeaux *et al.* [33] and our own analysis of their data revealed that only a subpopulation of SciP ChIP-seq peaks (47%) contain the TTAACAT motif. However, we found that 88% of the SciP ChIP-seq peaks contain a SciP binding motif. Of the 47% that contain a CtrA binding motif, 33/35 of these also contain the SciP binding motif. The SciP motif used in this study is based on previously reported direct footprints to DNA, ChIP-chip analysis and microarray analysis reported in Tan et al. [8], which is in agreement with a motif previously identified for a cohort of genes expressed at the same time in the cell cycle by McGrath *et al.* [25]. The SciP binding motif correlates with a larger percentage of the SciP ChIP-seq peaks than the CtrA motif. Both SciP half and CtrA half (TTAACAT) motifs are present in the CtrA promoter foot-printed by Tan *et al.* [8]. However, only protection of the SciP sites was observed with purified SciP. We suggest that the Fumeaux *et al.* ChIP-seq data analysis [33] missed many of the SciP motifs in the presence of stronger CtrA motif signals from peaks including SciP and CtrA co-regulated promoters. Indeed, the peaks of the Fumeaux *et al.* [33] SciP and CtrA ChIP-seq signal match the positions of their respective binding motifs (S8 Fig.).

### Combinatorial regulation of cell cycle activated promoters

We found that 57% of the cell cycle-regulated TSSs have upstream binding sites for known cell cycle transcriptional regulators (Fig. 2B-C, S4 Fig., S5-S12 Dataset) whose activity and protein levels oscillate in time [1], [4]. While 199 cell cycle regulated TSSs contain a single regulatory factor binding site, 135 have binding sites for 2 or more of these factors (S13 Dataset). We have not yet identified the regulatory factors (and their binding sites) that control the other 43% of the cell cycle regulated TSSs but we expect to find that the activity of these promoters are controlled by a second layer of regulatory factors that are turned on by the core circuit (Fig. 2B). Co-regulation among the 5 cell cycle master regulators acts to tune the timing of cell cycle transcription profiles, and we expect to find equally complex timing regulation in every genetic regulatory sub-system.

DnaA directly controls the initiation of DNA replication by binding to the origin of replication [67] and it also serves as a transcription factor for a large complement of cell cycle regulated genes [10]. We identified 77 cell cycle-regulated TSSs with a DnaA binding motif. Additionally, from our analysis of the ChIP-seq data of [26] we found 94 cell cycle-regulated TSSs with enriched upstream binding of GcrA, a transcriptional activator [9]. In contrast to to the temporal expression of CtrA-controlled genes, DnaA and GcrA regulated TSSs have a multitude of different cell cycle profiles with lower levels of activation (S6 Fig.). This increase in profile diversity is likely due to an increased number of co-regulated promoters for genes controlled by both DnaA and GcrA. Additionally, when DnaA and GcrA binding sites are combined with a CtrA binding site, the transcriptional profile appears to be dominated by the activity of CtrA.

Finally, we identified CcrM methylation sites within 96 cell cycle-regulated TSSs (Fig. 4A–B). We found cell cycle-controlled TSSs with GANTC motifs in these promoter regions, such as the *ctrA* P1 promoter which is activated by hemi-methylation, in agreement with a previous report of the control of this gene by methylation state of its promoters [37]. A cluster of these TSSs, located between −35 and −10 of the promoter region (Fig. 4B–C Red), are activated between 40 and 60 minutes. Another cluster of TSSs, with GANTC sites positioned outside of the −10 and −35 region, exhibit minimal TSS levels between 40 and 60 minutes (Fig. 4B–C Blue). While the underlying mechanism of methylation dependent transcription regulation in *Caulobacter* is unknown, the GcrA transcription factor has been implicated in the control of some GANTC containing promoters [68]. However, we find that only 31% of cell cycle regulated TSSs enriched for GcrA binding (from the published ChIP-seq data from [26]) also contain GANTC methylation sites within 50 bp upstream of the TSS (Fig. 2C, S4 Fig.). Additionally, the promoters of only 11 genes found to bind GcrA by ChIP-seq are differentially expressed in GcrA or CcrM depletion arrays [69].

Cumulatively, the newly-revealed complexity of transcriptional regulation that drives the *Caulobacter* cell cycle, coupled to multiple modes of post transcriptional regulation [13] has opened up new avenues of bacterial systems control architecture. The known *Caulobacter* cell cycle regulatory circuit, composed of four transcription factors and a DNA methyltransferase, drives stage specific expression of a majority of cell cycle-regulated promoters [2]. We found multiple examples of co-regulation using these five master regulators of the cell cycle regulatory circuit, and many of these examples involve essential genes and essential regulatory regions of the genome [22] (S13 Dataset). The presence of each master regulator is restricted to specific times in the cell cycle [8], [70], so that there are multiple cell cycle stage-specific regulatory options to tune the timing of transcription. The core *Caulobacter* cell cycle transcription regulatory network is conserved in a majority of α-proteobacteria [2], [60] and has been shown to be integrated with other regulatory pathways such as quorum sensing [71], plant symbiosis [61], [72], gene transfer agent production [73], [74], host cell infection [75], [76], and motility [73]. These α-proteobacterial species have adapted their cell cycle control circuits to their specific biological niches. Negative feedback by antisense regulation and the existence of many promoters with multiple TSSs that appear to modulate the cell cycle stage of mRNA production identifies yet other levels of regulatory control.

## Materials and Methods

### Cell growth and DNA sequencing library preparation

All 5′ Global RACE experiments were performed with *C. crescentus* strain CB15N (NA1000) [77]. Cells were grown to OD_600_ 0.4 in M2G [78] and synchronized using standard procedures [77]. Aliquots were taken at 20 min intervals over ∼1 cell cycle (140 min), pelleted and immediately frozen in liquid nitrogen. Total RNA was isolated using Trizol Plus kit (Invitrogen) according to the manufacturer protocol. Ten micrograms of total RNA isolated from each aliquot of cells were treated with MICROBExpress (Ambion) to remove ribosomal RNA following the manufacturer protocol. Ribosomal RNA depleted total RNA was treated with 10 U of Tobacco Acid Pyrophosphatase (TAP) (epicentre) for 1.5 hours at 37°C. In addition, two sequencing libraries were prepared from an unsynchronized cell population grown to OD_600_ 0.4 in M2G, one of which was not treated with TAP. Purified RNA was ligated with 10 pmol of RNA adapter (5'-ACACUCUUUCCCUACACGACGCUCUUCCGAUCU-3') using 25 U of T4 RNA Ligase 1 (New England BioLabs) for 12 h at 16°C. cDNA was then generated from purified RNA using SuperScript II Reverse Transcriptase (Invitrogen) with 10 pmol of primer (5'-CTCGGCATTCCTGCTGAACCGCTCTTCCGATCTNNNNNN-3') following standard manufacturer protocol then treated with 1 Unit of RNase H (Invitrogen) for 20 min at 37°C. cDNA was PCR amplified for 12 cycles using primers (5'-AATGATACGGCGACCACCGAGATCTACACTCTTTCCCTACACGACGCTCTTCCGATCT-3', 5'-CAAGCAGAAGACGGCATACGAGATCGGTCTCGGCATTCCTGCTGAACCGCTCTTCCGATCT-3'). DNA was size selected from 100 bp to 300 bp using agarose gel-electrophoresis and purified using QIAquick Gel Extraction Kit (Qiagen). 100ng of DNA was then treated with duplex-specific nuclease (evrogen) to remove ribosomal cDNA following standard manufacturer protocol and PCR amplified for 10 cycles.

### Read mapping and normalization

Sequencing reads were mapped using Bowtie version 0.12.7 [16] to the *C. crescentus* NA1000 reference sequence (Genbank: NC_011916). All valid alignments were made, and only reads that mapped to a unique location were used with no mismatches allowed. Sequencing reads were normalized by the total number of non-ribosomal RNA reads for comparison. +TAP and -TAP reads were normalized with relation to each other, and reads from synchronized cells were normalized with relation to each other.

### TSS identification

To differentiate between reads initiating from TSS and RNA processing sites, thirty-four previously biochemically characterized TSS with a minimum normalized read value of at least 30 in the +TAP library were used as a positive control, and twenty-four tRNA processing sites (*n* = 24) with a minimum normalized read value of at least 30 in the -TAP library were used as a negative control. The natural log value of the ratio (*θ*) between the number of sequencing reads obtained at the 5′ ends of positive and negative controls in +TAP/-TAP libraries was calculated. The mean and standard deviation of *θ* obtained for positive and negative controls were obtained (Welch two sample *t*-test, df  = 41.2, *p*-value  = 1.3 e−11, S1 Fig.). To minimize false-positives or Type I errors in TSS prediction, we set α = 2.3%, corresponding to *θ*>0.26. An increase of >35% of RNA-seq coverage [13] in a 38 bp window upstream compared to downstream of the TSS was also required.

The distance of mapped 5′ read locations upstream to CDSs in the *Caulobacter* genome was then determined. Reads that are mapped within 300 bp upstream of annotated CDSs or on the first base of the start codon were categorized as upstream promoters (category “P”); those within 300 bp upstream that are inside the upstream CDS but reside within the last 30% of the CDS if the CDS is longer than 600 bp or within the last 30% of the CDS if the CDS is shorter than 600 bp are categorized as internal upstream promoters (category “IP”). If the upstream CDS is oriented in the opposite direction as the TSS, then the TSS is categorized as an antisense upstream promoters (category “AP”). Unless already categorized as IP or AP for another CDS, reads that are mapped inside CDSs are categorized as internal promoters (category “I”); if oriented in the opposite direction of the CDS, the TSS is categorized as an antisense promoter (category “A”). All other reads were categorized as non-coding TSS (category “N”).

The total number of reads corresponding to each CDS as obtained from the +TAP library was calculated for both the plus and minus strands. Upstream promoter locations categorized as P, AP, or IP with reads contributing more than 10% of the total reads in the same orientation for a single CDS were kept. Antisense or internal promoter locations (A or I) with reads contributing more than 25% were kept. If multiple reads were mapped within a distance of ≤5 bp, the reads were summed and placed at the position with the highest number of reads in the +TAP library. TSS with ≥2 normalized reads in at least one of the cell cycle time points were kept for categories (P, AP, or IP); TSS with ≥10 normalized reads in at least one of the cell cycle time points were kept for other categories. Some TSSs categorized as N were actually upstream promoters for genes with unusually long 5′ UTRs. All TSSs categorized as N were manually curated to ensure accuracy.

### Algorithm to define cell cycle-regulated TSS

On the cell cycle expression profile of each TSS, Fourier coefficients (*coeff 0-7*) were calculated using normalized reads obtained from synchronized cells. The maximum and minimum normalized read values (defined as *a* and *b* respectively) from time 0 min to time 140 min were also determined. Cell cycle-regulated TSS are those that meet the following three criteria: 1.) *coeff1/(coeff1+ coeff2 + coeff3 + coeff4) ≥ 0.35* 2.) *ln(a) - ln(b) ≥ 1.1* 3.) *a ≥ 20.*

### Identifying regulatory factor binding sites

Regulatory binding sites were identified from groups of promoter sequences upstream of temporally clustered TSSs using MEME [79] and the position of the 5′ nucleotide of the conserved motifs are numbered relative to the TSS sites. The RpoD motif was obtained by searching within 50 bp upstream of constitutively active TSSs. Cell cycle-regulated TSSs were clustered (k-means, 15 clusters) by normalized cell cycle profile where the maximum of normalized read value within time 0–140 min is equal to 1. From the resulting clusters, up to 100 bp of upstream sequences were used to search for enriched CtrA, SciP, SigT, CcrM, and RpoN motifs using MEME. DnaA binding sites from [10] were used to generate a position weight matrix (PWM) to search for DnaA motifs within 100 bp upstream of cell cycle-regulated TSSs using FIMO (*p*-value setting: <0.001) [80]. To search for GcrA binding, we searched for ≥3 fold ChIP-seq enrichment within 50 bp upstream of cell cycle regulated TSSs, using ChIP-seq data from [26]. The enrichment was calculated relative to the average coverage across the genome.

### Strain generation andβ-galactosidase assays

75 bp of the upstream sequence of 36 TSSs including primary (P), anti-sense (A), and intergenic non-coding (N) TSSs (see S3 Dataset) were cloned into the Bgl II and Xho I restriction sites of vector pNJH185 [81], resulting in transcriptional fusions with the *lacZ* reporter gene. For the *ctrA* antisense promoter, 200 bp of upstream sequence was cloned between the Bgl II and XhoI sites. For promoter constructs mutating the SciP binding motif, 105 bp of upstream DNA were cloned between the Bgl II and Xho I sites (S9 Fig.). In each other case, the Bgl II site is upstream and the Xho I site is downstream of the +5 site of the RNA transcript. These 36 constructs and a pNJH185 empty vector control were introduced into *Caulobacter crescentus* NA1000 cells by electroporation. The LacZ activity of all constructs was measured using mid-log phase NA1000 cell cultures grown in minimal media and according to standard ONPG based β-galactosidase assays. Results in S3 Dataset represent the average of three independent measurements for each strain.

## Supporting Information

S1 Fig Ratio of reads from +TAP/-TAP sequencing libraries. Histogram of *θ* (x-axis), the natural log ratio of normalized reads between +TAP/-TAP sequencing libraries for biochemically validated TSS (S2 Dataset) where +TAP reads > 25 and difference ≤ 5 bp (blue, n = 34, mean  =  1.71, sd  =  1.26, S2 Dataset) and for the 5′ process sites of tRNAs where -TAP reads >25 (red, n = 24, mean  = −0.52, sd  = 0.35). Normality of distributions are verified by Chi square goodness-of-fit test (*p*<0.05). Welch two sample t-test: df  = 41.2, *p*-value  = 1.3 e −11. The threshold for TSS determination is set at *θ* >0.26.
(TIF)

S2 Fig Identification of the major sigma factor binding motif. RpoD, σ^73^, −35 and −10 binding motif (*e*-value  = 1.3 e −1663) identified by searching in genomic regions 45 bp upstream of 1,667 TSS using MEME [79], see S4 Dataset for the full MEME result summary. Histogram of the distance (bp) relative of the TSS in which the 5′ nucleotide of the motif is found (Right).
(TIF)

S3 Fig Global comparison of 5′ Global RACE TSS levels with microarray levels of cell cycle regulated genes with a single TSS. Heat map of the 5′ RACE generated cell-cycle TSS levels (left) with those generated by tiling arrays (right) [25]. A total of 206 genes/operons with a single TSS and average microarray probe intensity of greater than 0.2 are displayed for comparison. Each tiling array sample were taken 12 minutes apart, however, the 72 min sample failed and is omitted from the heat map [25].
(TIF)

S4 Fig Pairwise combinations of master regulators in cell cycle-regulated promoter regions. Number of cell cycle-regulated TSSs with pairwise combinations of DnaA, CtrA, SciP, CcrM binding motifs or a >3 fold enrichment of GcrA Chip-seq signal [26] in upstream promoter regions. Many promoter regions contain more than two TF binding sites so that the indicated number of total TSSs in panel B is always lower than the sum of the pairwise combinations. The number of cell cycle-regulated TSSs for each component of the cell cycle control circuit including the number of promoter regions containing each pairwise combination.
(TIF)

S5 Fig TSSs activated by cell cycle-regulated sigma factors. SigT binding motif GGAAC-N16-CGTT ([27]) (n = 26, *e*-value  = 1.9 e −39) and RpoN binding motif GGCNC-N4-CTTGC ([28]) (n = 33, e*-*value  = 4.3 e−19) within 100 bp upstream of cell cycle-regulated of TSS. Normalized TSS levels as a function of the cell cycle is shown on the right. Cell cycle TSS profile values (y-axis) indicate the fraction of reads relative to the maximum obtained during the cell cycle, and error bars represent standard error. The group of 33 TSS with an enriched RpoN motif can be divided into two classes based on average cell cycle profiles shown in red and green. Histograms show the distances from which the SigT and RpoN motifs are found relative to the TSS.
(TIF)

S6 Fig Cell cycle TSS levels of all combinations of master regulator binding motifs. Average TSS levels with individual or pairwise combinations of DnaA, CtrA, SciP, CcrM binding motifs or a >3 fold enrichment of GcrA Chip-seq signal [26] in upstream promoter regions. Error bars indicate standard error. See S4 Fig. for number of TSS in each pairwise combination. The numbers of TSS regulated by only one master regulator (no combinatorial regulation) are: DnaA, 29; GcrA, 34; CtrA, 89; SciP, 7; CcrM, 40.
(TIF)

S7 Fig 5′ global RACE sequencing library preparation. Samples of total RNA (rRNA depleted) were either treated with tobacco acid pyrophosphatase (TAP) and ligated with the 5′ sequencing adapter or taken directly to ligation with a 5′ sequencing adapter. TAP treatment converts RNA 5′ tri-phosphate groups to 5′ mono-phosphate groups and subsequently ligated to a 5′ RNA adapter. Ligated RNAs were purified, reverse transcribed, PCR amplified and DSN treated for an additional round of rRNA removal.
(TIF)

S8 Fig CtrA and SciP ChIP-seq signal peaks over their respective binding motifs. ChIP-seq data for SciP and CtrA from [33] were mapped to the genome using bowtie, and the average normalized ChIP-seq signal is plotted for each TSS containing a SciP binding motif (from S8,S9 Dataset). SciP ChIP-seq signal (blue) is highest upstream of the CtrA ChIP-seq signal (red) corresponding to the positions of the respective binding motifs (Fig. 3).
(TIF)

S9 Fig Mutation of the SciP binding motif leads to increased promoter activity. (A) LacZ promoter fusions were generated spanning the −100 to +5 of SciP motif containing promoter regions. Promoter constructs were inserted into pNJH185 between the BglII and XhoI sites. Plasmids were then sequence verified, transformed into strain NA1000, grown to mid-log phase in M2G media, and assayed for LacZ activity. The average and standard error for three independent experiments is plotted. (B) Sequences of each of the promoter fusions assayed. Mutations of the SciP site were designed to disrupt the entire binding motif.
(TIF)

S10 Fig Cell cycle timing of CtrA promoters co-regulated with MucR 1/2. The cell cycle timing of the average TSS levels for promoters containing CtrA binding motifs encoded in the presence or absence of ChIP-seq peaks of MucR 1/2 as determined in [33].
(TIFF)

S1 Dataset List of TSSs identified.
(XLSX)

S2 Dataset Comparison of previous biochemically determined TSSs with global 5' RACE.
(XLSX)

S3 Dataset β-galactosidase (beta-gal) verification of TSSs identified by global 5′ RACE by inserting the 75 bp sequence upstream of TSS in front of *lacZ*. A previously made construct was used for beta-gal assay of the *ctrA* (*CCNA_03130*) antisense TSS where 200 bp of upstream sequence was inserted in front of *lacZ*.
(XLSX)

S4 Dataset Cell cycle-regulated TSSs with enriched RpoD (σ^73^) motif (S2 Fig.).
(XLSX)

S5 Dataset Cell cycle-regulated TSSs with enriched CtrA full motif (Fig. 3A).
(XLSX)

S6 Dataset Cell cycle-regulated TSSs with enriched CtrA half motif repressor (Fig. 3A).
(XLSX)

S7 Dataset Cell cycle-regulated TSSs with enriched CtrA half motif activator (Fig. 3A).
(XLSX)

S8 Dataset Cell cycle-regulated TSSs with enriched SciP full motif (Fig. 3B).
(XLSX)

S9 Dataset Cell cycle-regulated TSSs with enriched SciP half motif (Fig. 3B).
(XLSX)

S10 Dataset Cell cycle-regulated TSSs with DnaA motif.
(XLSX)

S11 Dataset Cell cycle-regulated TSSs with methyltransferase CcrM methylation motif within (1–50 bp) of upstream promoter regions (Fig. 4).
(XLSX)

S12 Dataset Cell cycle-regulated TSSs with enriched GcrA Chip-seq peaks.
(XLSX)

S13 Dataset Cell cycle-regulated TSSs with combinatorial regulation by DnaA, GcrA, CtrA, SciP, or CcrM (Fig. 2C, S4 Fig.).
(XLSX)

S14 Dataset Cell cycle-regulated TSSs with enriched SigT motif (S5 Fig.).
(XLSX)

S15 Dataset Cell cycle-regulated TSSs with enriched RpoN motif (S5 Fig.).
(XLSX)

S16 Dataset Putative antisense TSSs identified by global 5′ RACE without secondary verification.
(XLSX)

S17 Dataset Cell cycle-regulated antisense TSSs with levels peaking at different times than the cell cycle-regulated sense TSS.
(XLSX)

S18 Dataset TSSs of annotated non-coding ncRNAs (excluding rRNA and tRNA genes).
(XLSX)

S19 Dataset CDS with multiple primary TSSs.
(XLSX)

S20 Dataset Cell cycle-regulated TSSs inside operons.
(XLSX)
