# Supplementary figures and images for: The Global Regulatory Architecture of Transcription during the Caulobacter Cell Cycle
Source: PLoS Genet. 2015 Jan 8;11(1):e1004831. doi: 10.1371/journal.pgen.1004831 (PMC4287350; doi:10.1371/journal.pgen.1004831)

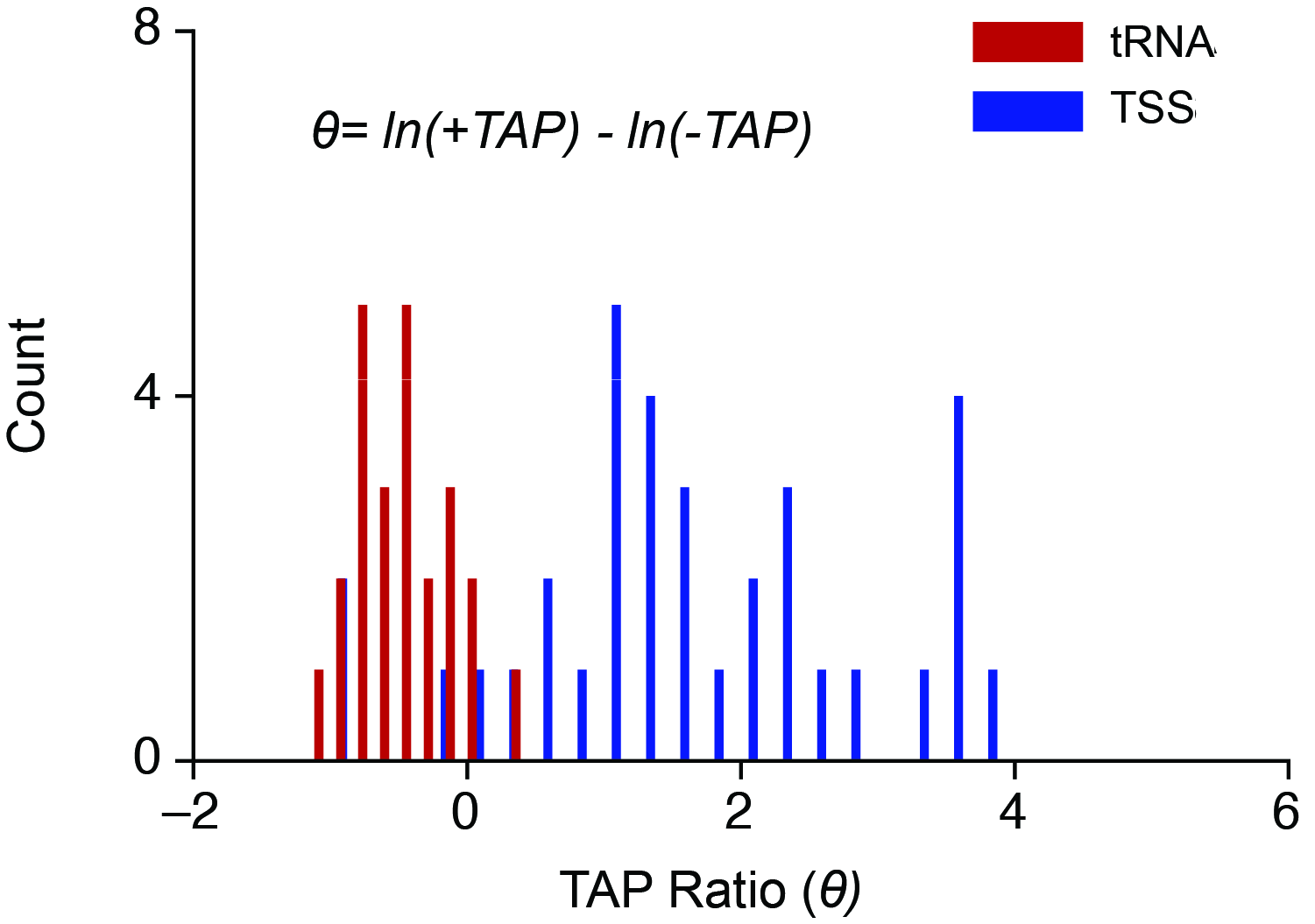

Supplement: S1 Fig — Ratio of reads from +TAP/-TAP sequencing libraries. Histogram of θ (x-axis), the natural log ratio of normalized reads between +TAP/-TAP sequencing libraries for biochemically validated TSS (S2 Dataset) where +TAP reads > 25 and difference ≤ 5 bp (blue, n = 34, mean = 1.71, sd = 1.26, S2 Dataset) and for the 5′ process sites of tRNAs where -TAP reads >25 (red, n = 24, mean = −0.52, sd = 0.35). Normality of distributions are verified by Chi square goodness-of-fit test (p<0.05). Welch two sample t-test: df = 41.2, p-value = 1.3 e −11. The threshold for TSS determination is set at θ >0.26. (TIF) [file pgen.1004831.s001.tif]

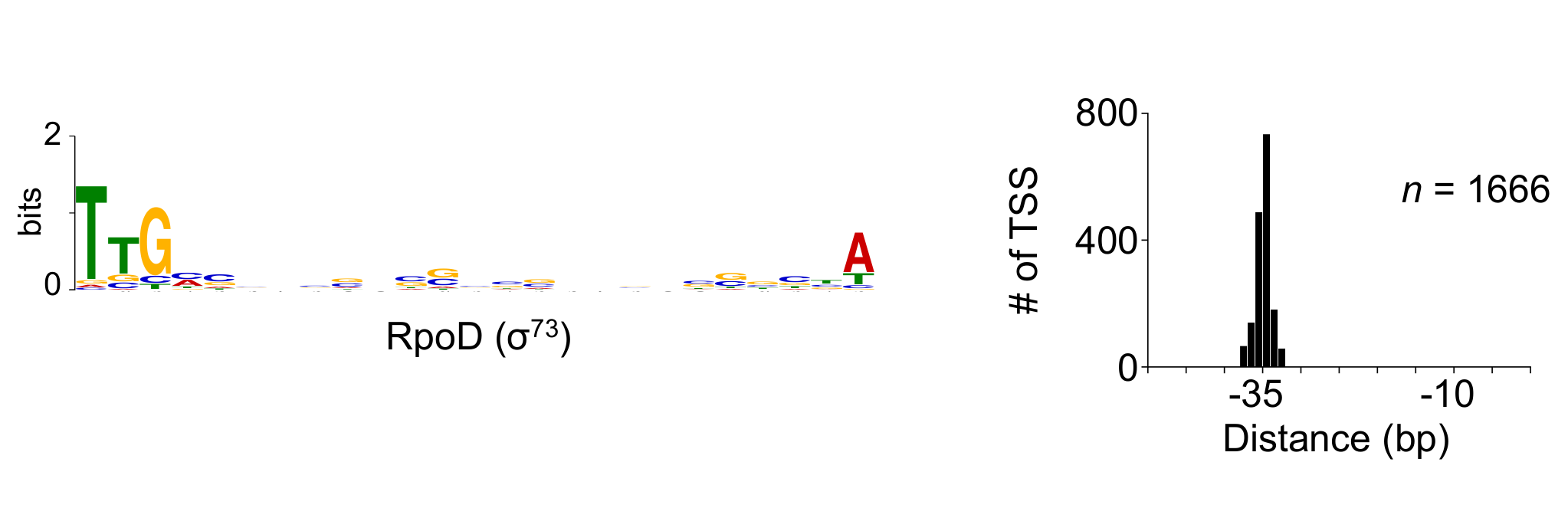

Supplement: S2 Fig — Identification of the major sigma factor binding motif. RpoD, σ73, −35 and −10 binding motif (e-value = 1.3 e −1663) identified by searching in genomic regions 45 bp upstream of 1,667 TSS using MEME [79], see S4 Dataset for the full MEME result summary. Histogram of the distance (bp) relative of the TSS in which the 5′ nucleotide of the motif is found (Right). (TIF) [file pgen.1004831.s002.tif]

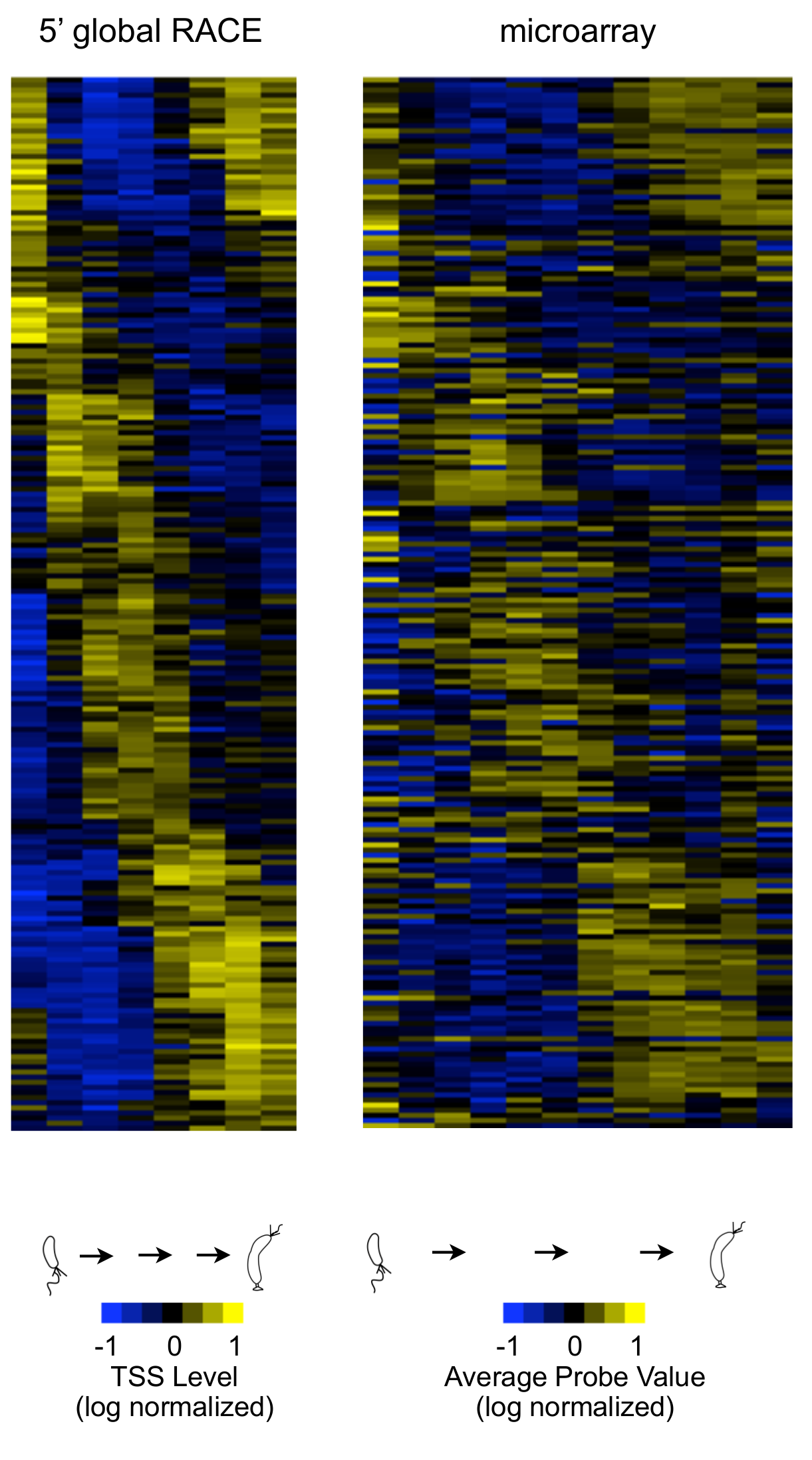

Supplement: S3 Fig — Global comparison of 5′ Global RACE TSS levels with microarray levels of cell cycle regulated genes with a single TSS. Heat map of the 5′ RACE generated cell-cycle TSS levels (left) with those generated by tiling arrays (right) [25]. A total of 206 genes/operons with a single TSS and average microarray probe intensity of greater than 0.2 are displayed for comparison. Each tiling array sample were taken 12 minutes apart, however, the 72 min sample failed and is omitted from the heat map [25]. (TIF) [file pgen.1004831.s003.tif]

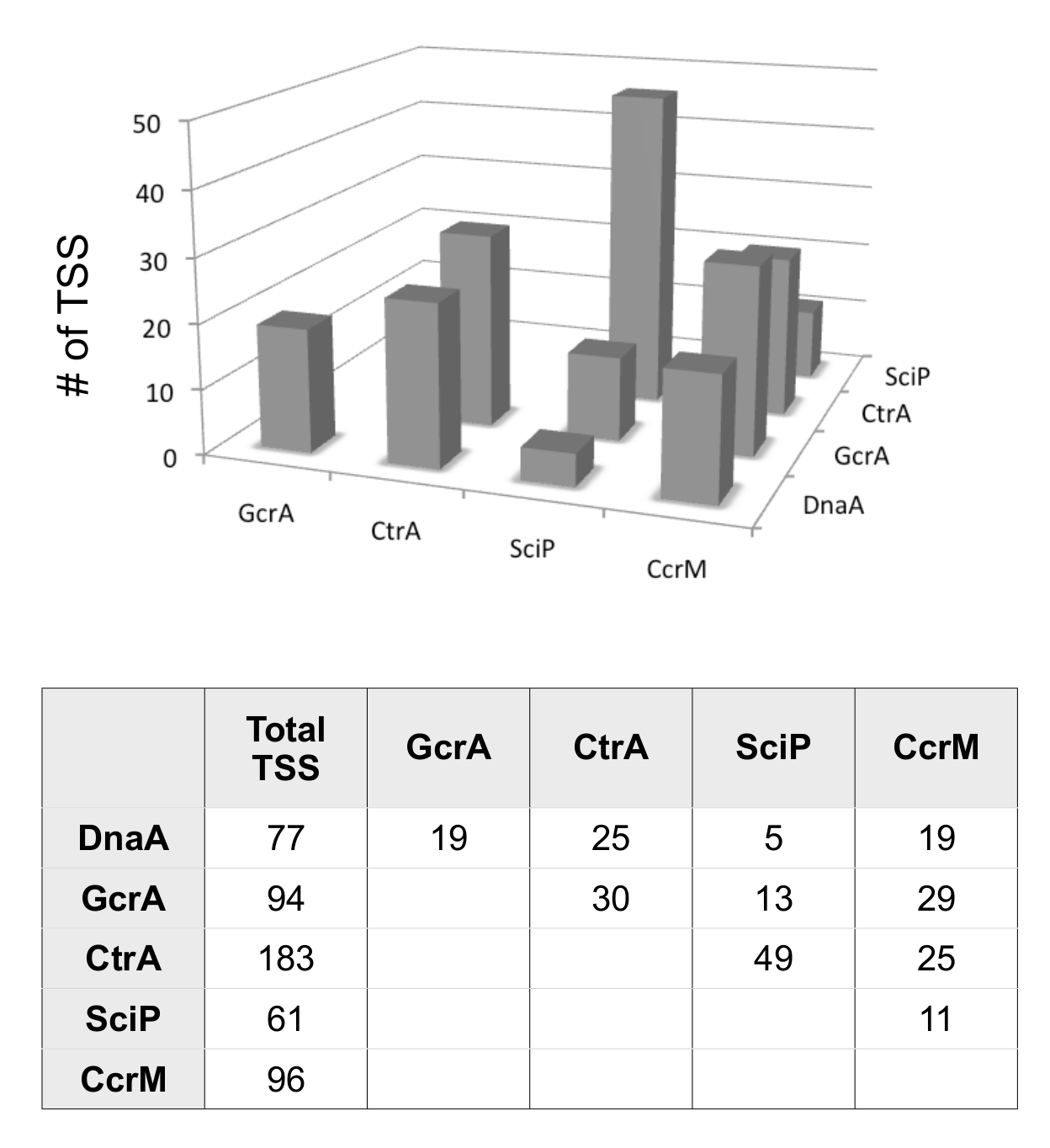

Supplement: S4 Fig — Pairwise combinations of master regulators in cell cycle-regulated promoter regions. Number of cell cycle-regulated TSSs with pairwise combinations of DnaA, CtrA, SciP, CcrM binding motifs or a >3 fold enrichment of GcrA Chip-seq signal [26] in upstream promoter regions. Many promoter regions contain more than two TF binding sites so that the indicated number of total TSSs in panel B is always lower than the sum of the pairwise combinations. The number of cell cycle-regulated TSSs for each component of the cell cycle control circuit including the number of promoter regions containing each pairwise combination. (TIF) [file pgen.1004831.s004.tif]

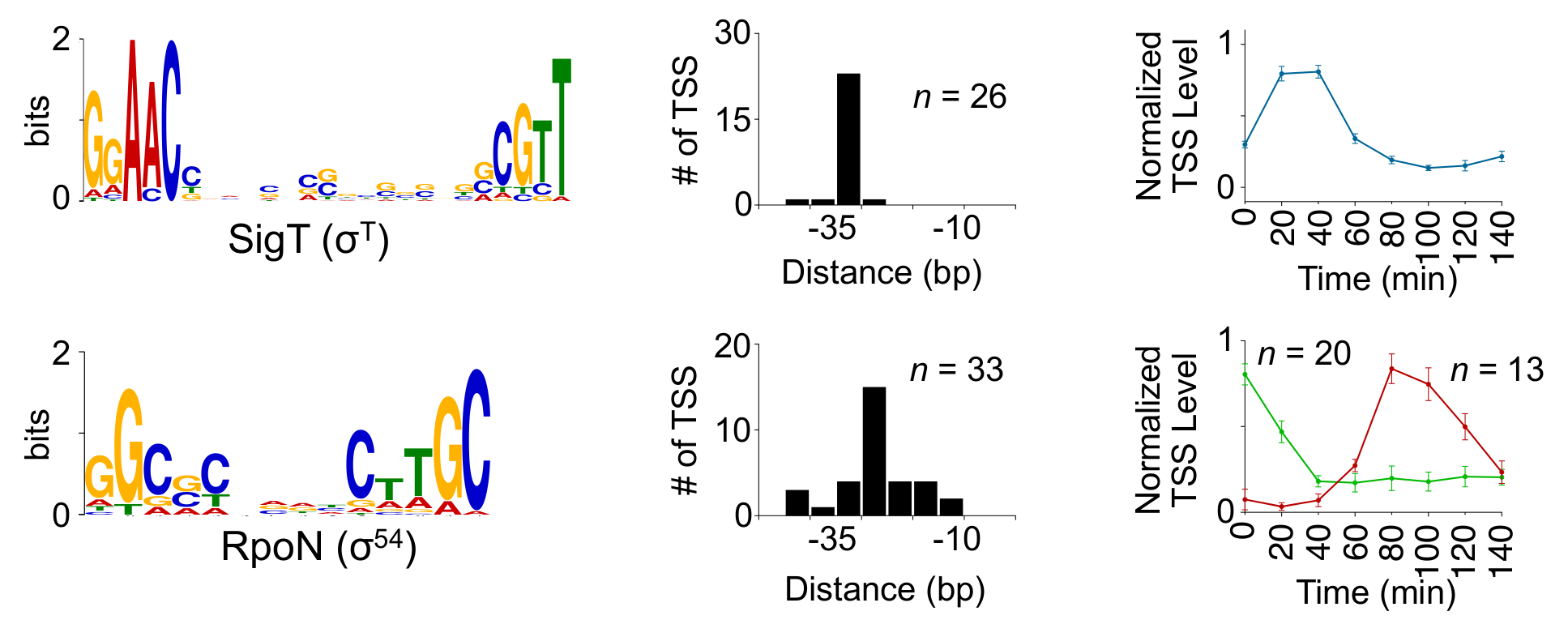

Supplement: S5 Fig — TSSs activated by cell cycle-regulated sigma factors. SigT binding motif GGAAC-N16-CGTT ([27]) (n = 26, e-value = 1.9 e −39) and RpoN binding motif GGCNC-N4-CTTGC ([28]) (n = 33, e-value = 4.3 e−19) within 100 bp upstream of cell cycle-regulated of TSS. Normalized TSS levels as a function of the cell cycle is shown on the right. Cell cycle TSS profile values (y-axis) indicate the fraction of reads relative to the maximum obtained during the cell cycle, and error bars represent standard error. The group of 33 TSS with an enriched RpoN motif can be divided into two classes based on average cell cycle profiles shown in red and green. Histograms show the distances from which the SigT and RpoN motifs are found relative to the TSS. (TIF) [file pgen.1004831.s005.tif]

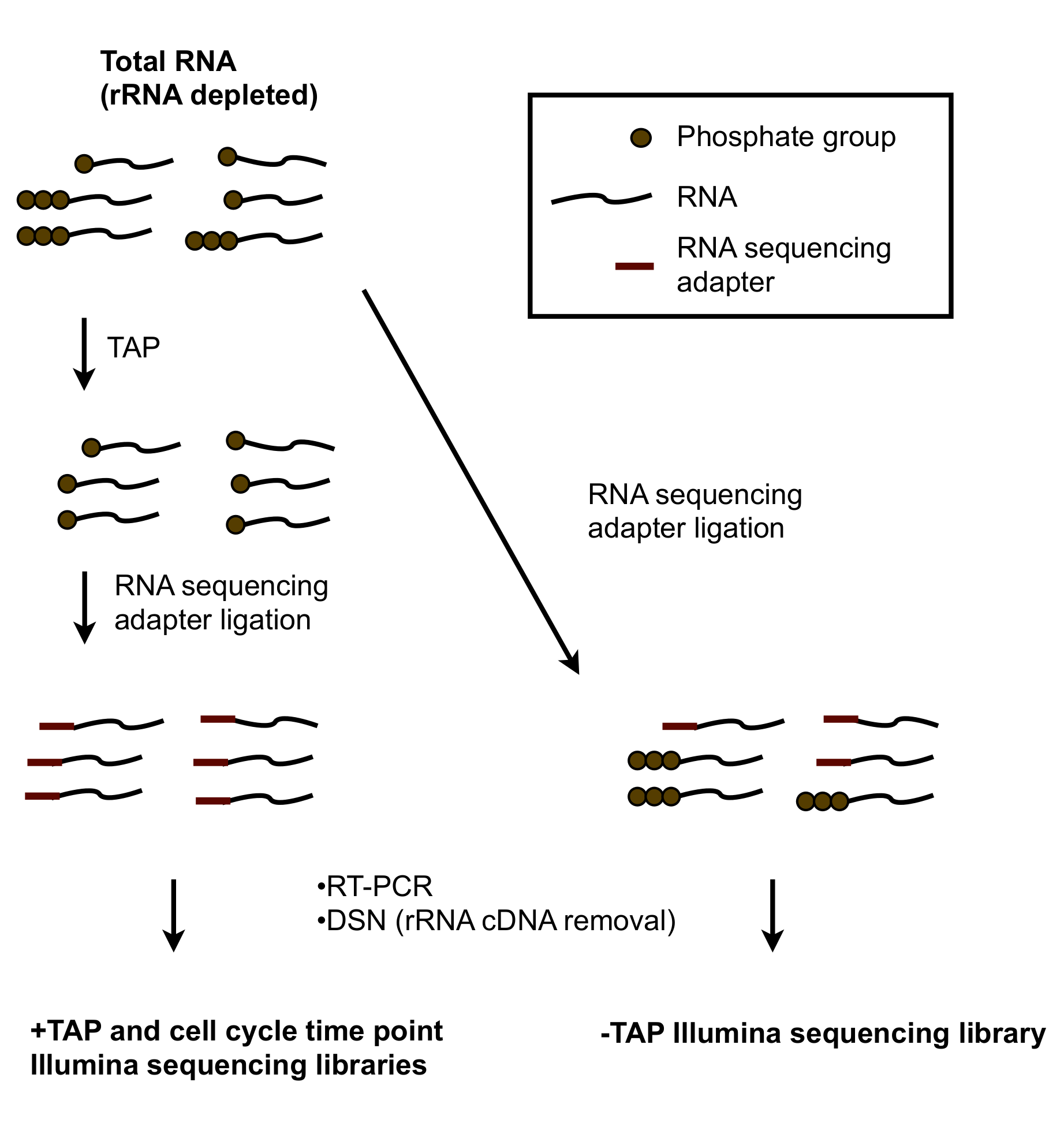

Supplement: S7 Fig — 5′ global RACE sequencing library preparation. Samples of total RNA (rRNA depleted) were either treated with tobacco acid pyrophosphatase (TAP) and ligated with the 5′ sequencing adapter or taken directly to ligation with a 5′ sequencing adapter. TAP treatment converts RNA 5′ tri-phosphate groups to 5′ mono-phosphate groups and subsequently ligated to a 5′ RNA adapter. Ligated RNAs were purified, reverse transcribed, PCR amplified and DSN treated for an additional round of rRNA removal. (TIF) [file pgen.1004831.s007.tif]

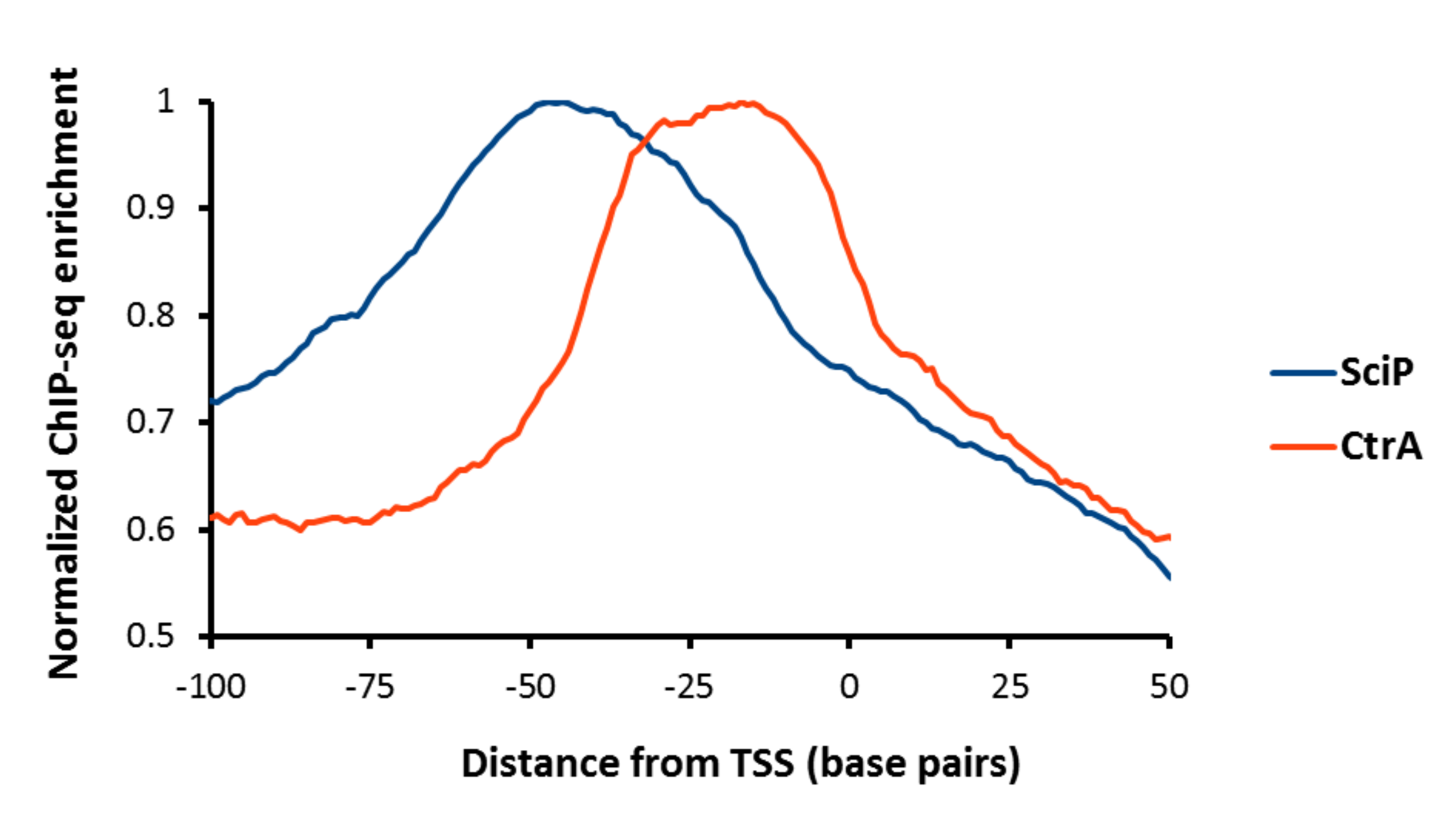

Supplement: S8 Fig — CtrA and SciP ChIP-seq signal peaks over their respective binding motifs. ChIP-seq data for SciP and CtrA from [33] were mapped to the genome using bowtie, and the average normalized ChIP-seq signal is plotted for each TSS containing a SciP binding motif (from S8,S9 Dataset). SciP ChIP-seq signal (blue) is highest upstream of the CtrA ChIP-seq signal (red) corresponding to the positions of the respective binding motifs (Fig. 3). (TIF) [file pgen.1004831.s008.tif]

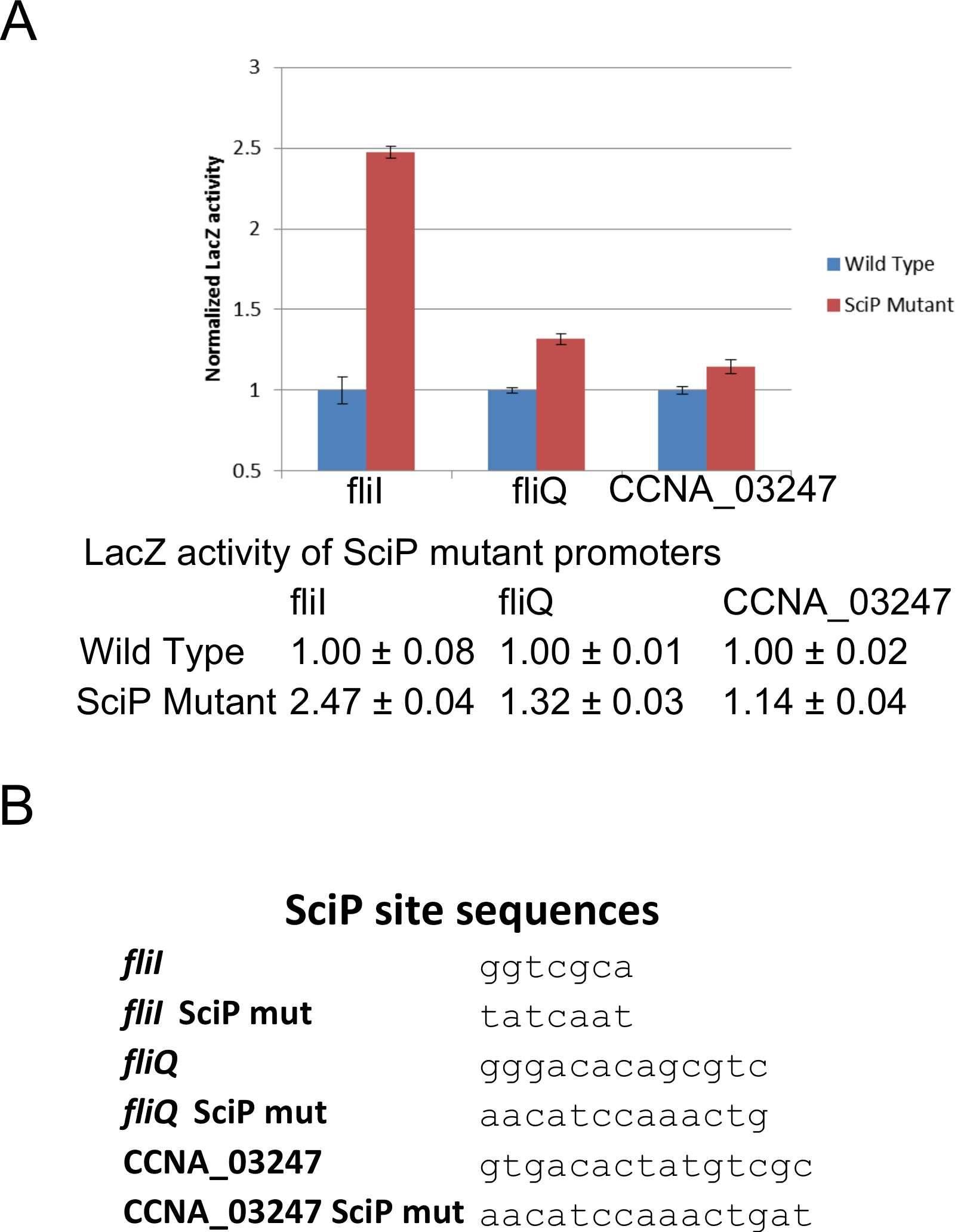

Supplement: S9 Fig — Mutation of the SciP binding motif leads to increased promoter activity. (A) LacZ promoter fusions were generated spanning the −100 to +5 of SciP motif containing promoter regions. Promoter constructs were inserted into pNJH185 between the BglII and XhoI sites. Plasmids were then sequence verified, transformed into strain NA1000, grown to mid-log phase in M2G media, and assayed for LacZ activity. The average and standard error for three independent experiments is plotted. (B) Sequences of each of the promoter fusions assayed. Mutations of the SciP site were designed to disrupt the entire binding motif. (TIF) [file pgen.1004831.s009.tif]

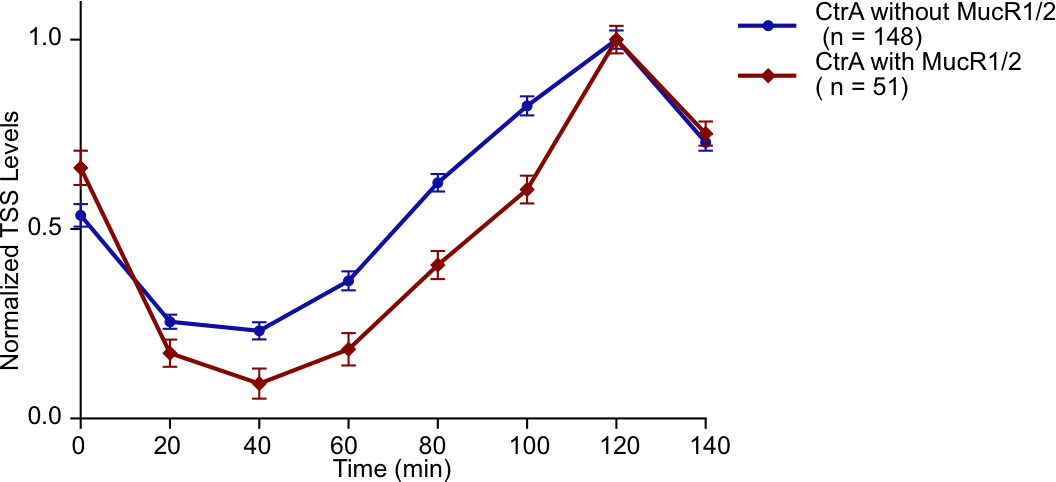

Supplement: S10 Fig — Cell cycle timing of CtrA promoters co-regulated with MucR 1/2. The cell cycle timing of the average TSS levels for promoters containing CtrA binding motifs encoded in the presence or absence of ChIP-seq peaks of MucR 1/2 as determined in [33]. (TIFF) [file pgen.1004831.s010.tif]
